# Li + HF and Li + HCl Reactions Revisited I: QCT Calculations and Simulation of Experimental Results

**DOI:** 10.1021/acs.jpca.3c03763

**Published:** 2023-08-14

**Authors:** Marta Menéndez, Ernesto Garcia, Manuel Lara, Pablo G. Jambrina, F. Javier Aoiz

**Affiliations:** † Departamento de Química Física, Facultad de Ciencias Químicas, Universidad Complutense de Madrid, 28040 Madrid, Spain; ‡ Departamento de Química Física, Universidad del País Vasco (UPV/EHU), 01006 Vitoria, Spain; § Departamento de Química Física Aplicada, Facultad de Ciencias, Universidad Autónoma de Madrid, 28039 Madrid, Spain; ∥ Departamento de Química Física, Facultad de Ciencias Químicas, Universidad de Salamanca, 37008 Salamanca, Spain

## Abstract

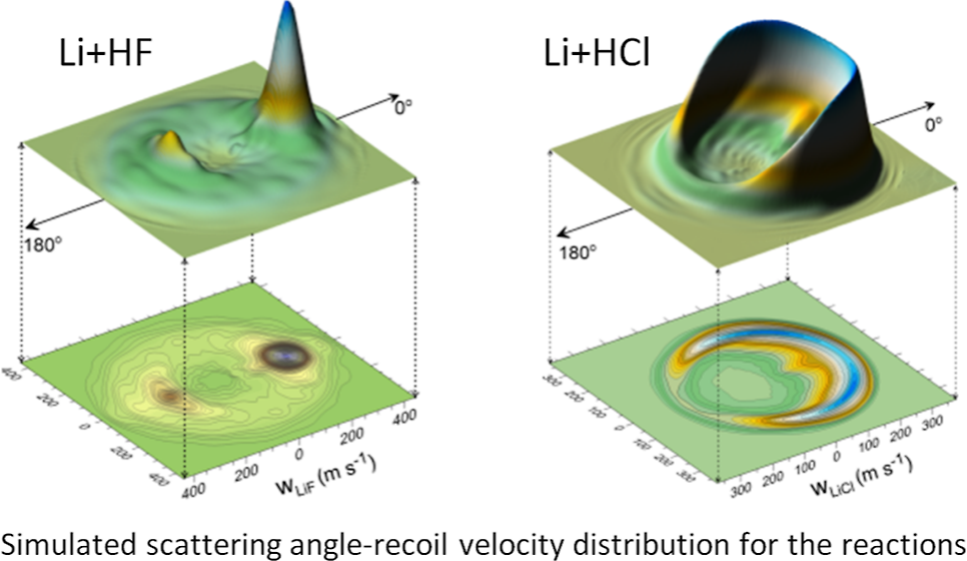

The Li + HF and Li + HCl reactions share some common features. They have the same kinematics, relatively small barrier heights, bent transition states, and are both exothermic when the zero point energy is considered. Nevertheless, the pioneering crossed beam experiments by Lee and co-workers in the 80s (Becker et al., *J. Chem. Phys.***1980,***73,* 2833) revealed that the dynamics of the two reactions differ significantly, especially at low collision energies. In this work, we present theoretical simulations of their results in the laboratory frame (LAB), based on quasiclassical trajectories and obtained using accurate potential energy surfaces. The calculated LAB angular distributions and time-of-flight spectra agree well with the raw experimental data, although our simulations do not reproduce the experimentally derived center-of-mass (CM) differential cross section and velocity distributions. The latter were derived by forward convolution fitting under the questionable assumption that the CM recoil velocity and scattering angle distribution were uncoupled, while our results show that the coupling between them is relevant. Some important insights into the reaction mechanism discussed in the article by Becker et al. had not been contrasted with those that can be extracted from the theoretical results. Among them, the correlation between the angular momenta involved in the reactions has also been examined. Given the kinematics of both systems, the reagent orbital angular momentum, , is almost completely transformed into the rotation of the product diatom, ***j***′. However, contrary to the coplanar mechanism proposed in the original paper, we find that the initial and final relative orbital angular momenta are not necessarily parallel. Both reactions are found to be essentially direct, although about 15% of the LiFH complexes live longer than 200 fs.

## 1 Introduction

After the seminal experiment of Datz and Taylor on the reactive system K + HBr,^1^ the crossed molecular beams (CMB) technique expanded rapidly and became extensively used to investigate the dynamics of exchange reactions of the type M + HX → MX + H, where X is a halogen atom, and M is an alkali. In this technique, particularly powerful for investigating gas-phase reaction dynamics under single-collision conditions,^2−4^ two supersonic beams are prepared with a precise velocity and made to collide at a specific angle. In principle, this allows for the selection of the collision energy, *E*_coll_, and the preparation of the internal quantum state of the reactants. Before the advent of lasers as a routine tool, product detection in CMB experiments was usually performed by electron-ionization mass spectrometry, which is a standard universal method. By using a rotatable detector, the angular distribution of the products can be determined, hence allowing the product number density to be obtained in the laboratory frame (LAB) as a function of the scattering angle and time-of-flight (TOF).^2^ The deconvolution of these data generates contour maps of the product flux as a function of the angle and recoil velocity in the center-of-mass (CM) frame, and hence a way to fully characterize the angular and velocity distributions of the products. The CMB experiments with mass-spectrometric detection, pioneered by Lee and co-workers in the 70s and 80s, burgeoned and became the standard technique to study a great variety of reactions as well as inelastic collisions.^5−10^ The advent of lasers paved the way for more accurate experimental techniques, in which the reagents were produced by photodissociation, allowing the preparation of a very narrow collision energy distribution. In addition, laser detection of the products by LIF,^11,12^ REMPI, Rydberg-tagging,^13^ and near-threshold ionization has made possible full state-to-state measurements. Currently, the standard technique is velocity-mapping ion imaging to measure with great accuracy state-resolved differential cross sections (DCSs).^14,15^ In addition, by using external fields or laser polarization, reagents can be aligned or oriented relative to the incoming relative velocity, *v*_r_, providing information on the steric requirements of the reaction and the possible control of the outcome of a reactive or inelastic collision.^16−20^ Also, over the last decade, variable-angle CMB setups have allowed to reduce *v*_r_(21) and hence to explore the dynamics of bimolecular collisions at very low collision energies.^22−26^ Stark and Zeeman decelerators can be included in one of the arms of the apparatus, leading to impressive control of the resolution of the kinetic energy of the beam. This has made it possible to tune collision resonances.^27^ Furthermore, the use of beams which intersect at very small angles (merged beams),^28−30^ and the advent of new coherent control techniques open up an exciting scenario where the cold and ultracold regimes can be explored with almost complete selection of translational, electronic, rovibrational, and even hyperfine selection of the state of the reactants.^31^

Among the CMB experiments performed by Lee and collaborators in the beginning of the 80s, they published the first experimental data on the Li + HF and Li + HCl systems.^32^ Using the *universal machine*, they determined reactive and non-reactive angular distributions in the LAB system and TOF spectra at different collision energies in the 0.095–0.378 eV range for the Li + HF reaction and in the 0.082–0.399 eV range for Li + HCl. The whole work constituted a milestone in the study of reactive collisions, and the detailed analysis of the experimental data provided invaluable information on the dynamics of the two reactions. Although these reactions share the same H + HL → HH + L (H = heavy, L = light) kinematics, the experiments revealed profound differences between them.

In the case of the Li + HF → LiF + H reaction for a collision energy, *E*_coll_ = 0.130 eV (3.0 kcal mol^–1^), the analysis of the scattering angle–recoil velocity distributions after transformation to the CM frame exhibits a near forward–backward symmetry, peaking slightly in the forward hemisphere. This was interpreted by the authors as evidence of the formation of a complex whose lifetime is expected to be comparable to the rotational period. At *E*_coll_ = 0.378 eV (8.7 kcal mol^–1^), the LiF product was predominantly scattered in the forward direction. It seemed reasonable that with increasing *E*_coll_, the formation of long-lived collision complexes would become unimportant. As for the energy disposal in the products derived from the CM LiF recoil velocity distribution, a significant fraction of the energy available to the products (55%) appeared as translational energy, *E*_T_′. At 0.378 eV, the fraction into translational energy was similar, although it was suggested that the fraction into vibrational energy might be somewhat larger. However, their analysis had a limitation: they assumed that the angle-velocity DCS in the CM frame could be cast as the separable product of the angular and recoil velocity (or product translational energy) distributions. This assumption is tantamount to conjecture that the angular distribution (the DCS) does not depend, or it does slightly depend, on the internal states of the LiF product. In addition, the absolute value of the total (integral) reactive cross section was estimated by comparing reactive scattering signals with small-angle elastic signals, whose absolute value was inferred theoretically from the calculated scattering elastic signal based on the van der Waals (vdW) long-range interaction. In spite of the assumptions involved in this deduction, it will be seen that their estimated values, of the order of 0.9 Å^2^, coincide almost exactly with those obtained in the present work and accurate quantum mechanical (QM) calculations.^33,34^ Another relevant discussion in the article by Becker et al.^32^ was the vector correlation between the initial, , and final, , orbital angular momenta since the final rotational angular momentum, ***j***′, must be parallel to  by kinematic constraint. They concluded that although ,  is likely to be parallel or antiparallel to the former, and therefore, the reaction would be mostly coplanar.

The study of the Li + HF reaction has been the subject of a large number of experimental and, above all, theoretical studies. In particular, Loesch and co-workers used the CMB technique to study the reaction in detail. They investigated the effect of the collision energy on the integral cross section (ICS) in the 0.025 to 0.376 eV range.^21,35^ To achieve *E*_coll_ < 0.100 eV, they varied the crossing angle between the Li and HF beams (down to 37°) and were able to show that the excitation function rises steeply as the collision energy decreases, which is consistent with a non-threshold reaction (if existed, it would be less than 0.020 eV), which is particularly interesting considering that the classical barrier is about 0.240 eV (0.08 eV taking into account the zero point energies). In a previous article, Loesch and Stienkemeier were able to demonstrate the presence of two vdW wells and proposed an empirical expression for them. It was found that the deeper vdW was of the order of 0.30 eV, in very good agreement with the accurate ab initio values of 0.240–0.266 eV.^36^ As we will see, this feature plays an important role in the reaction dynamics.

Loesch and co-workers also examined the effect of *E*_coll_(37) and of the HF rotational temperature on the DCS by changing the stagnation temperature of the nozzle and determining the HF state population by IR laser-induced fluorescence.^38^ The experimental LAB angular distribution (LAB AD) and TOF spectra were simulated with the results from quasiclassical trajectory (QCT) calculations at different collision energies and HF rotational states, showing a good agreement with the experimental data.^38^ The calculated polar maps (scattering angle–recoil velocity distributions) were similar to those deduced by Becker et al., showing a progressive relative enhancement of forward scattering with increasing collision energy. Although the calculations indicate a strong influence of rotation in the DCSs, it is difficult to disentangle its effect from that of the collision energy. The effect of the vibrational energy, exciting the HF molecules into (*v* = 1, *j* = 1), was also studied by Loesch, Stienkemeier, and co-workers using IR pumping with a tunable color center laser.^39,40^ The LAB ADs were simulated with the coupled angle-velocity DCSs calculated by QCT, showing a very good agreement with the experimental results. In a further step, they studied the effect of the HF (*v* = 1, *j* = 1) alignment on the reactivity, one of the first experiments in which the reagent was aligned with respect to the initial ***v***_r_.^40^ This was achieved by changing the direction of the laser polarization. To avoid the rapid depolarization due to the coupling of ***j*** with the nuclear spins, the excitation was carried out within a homogeneous electric field that resulted in the Stark splitting of the *j* = 1 state. The results indicate a strong influence of the perpendicular or parallel alignment with respect to the relative velocity on the measured LAB AD. Subsequent experiments by Höbel and Loesch refined the previous results by aligning the HF molecules along and perpendicular to ***v***_r_, as well as in the magic-angle direction with respect to ***v***_r_.^41^ The experimental results were reproduced almost quantitatively by QCT calculations using a formalism based on multipolar moment expansion.^42^

Several ab initio electronic energy calculations on the LiFH system have been carried out. The most accurate ab initio calculations^34,43−45^ of the stationary points predict a bent transition state (TS), but somewhat different values for the endoergicities and the location of the saddle points. They also predict two vdW complexes in the entrance channel and a shallower vdW complex in the product channel. An important conclusion of these studies is that the relative energies of the stationary points are very sensitive to the level of the theory. Most of the fits of the ab initio data^43,46−49^ were based on many-body expansions.^50^ However, the most recent PES was built using a neural network procedure.^34^

There are a large number of dynamics studies on the Li + HF → LiF + H reaction. Most of them focused on the calculation of the excitation function and the dependence of the reaction cross section on the collision energy. Only a few of them provide CM DCS. Such studies were carried out using quasiclassical trajectories,^38,39,51,52^ time-independent,^53,54^ and time-dependent^34,55,56^ full-dimensional quantum techniques. It is worth noting that dynamics results depend strongly on the PES, particularly at low translational energies.^33^

In contrast to the Li + HF reaction, the Li + HCl has received much less attention. Although the kinematics is similar for both reactions, the Li + HCl → LiCl + H reaction was found to be very different from that of Li + HF in the seminal article by Becker et al. The experimentally derived DCSs showed a predominance of forward scattering and no sign of backward–forward symmetry even at the lowest *E*_coll_ = 0.126 eV (2.9 kcal mol^–1^).^32^ The experimental product translational energy distributions at two collision energies (0.126 and 0.400 eV) give an average value of 70% of the total available energy. LAB ADs were also measured at 0.082 eV, which implies, if it exists, a lower threshold than this value. Based on the comparison between reactive and low-angle elastic scattering, the absolute cross sections for the LiCl formation were determined to be 27 Å^2^ at 0.126 eV and 42 Å^2^ at 0.399 eV. As will be shown, these values are far from those found in the QM calculations and the present QCT results.

In accordance with the scarcity of experimental data for the Li + ClH reaction, there is only a limited number of PESs based on ab initio calculations.^57−60^ The oldest ones^57,58^ were built with a limited number of ab initio data, but they already identified a bent saddle point for reaction. QCT calculations were carried out on these PESs to determine reaction cross sections, DCSs, and product’s translational energy release distributions as a function of the collision energy. The cross-section values obtained were definitively smaller than those derived by Becker et al., and the calculations also led to a narrower product’s translational energy distribution.^57,58^ The most recent PESs^59,60^ are based on tens of thousands of ab initio points calculated at the CASSCF–MRCI level and using very large basis sets. The PES of ref (59) exhibits a classical barrier of 0.163 eV and confirms a strongly bent TS. Using a time-dependent wave-packet QM (TD-WP QM) method, state-to-state ICSs as well as DCSs were calculated, showing a preference for forward scattering. Coupled-states TD-WP QM calculations have been recently carried out on this PES to determine the reaction probabilities and ICS and to study the vdW resonances.^61^ The most recent PES was constructed by Tan et al. (hereinafter TZYGL PES)^60^ interpolating by three-dimensional cubic splines over more than 36,000 ab initio points at the CASSCF–MRCI level using a polarized valence quintuple zeta (aug-cc-pV5Z) basis set. In the same article, TD-WP Coriolis coupled calculations (but with a limited number of helicity projections) were performed to calculate the excitation function up to 0.6 eV of collision energy for initial rotational states *j* = 0, 1, and 2. These results confirmed that the cross sections deduced by Becker et al. were largely overestimated. It must be pointed out that the two most recent PESs exhibit vdW wells in both the entrance and exit channels. The topography of the surfaces and the influence of the long-range attractive potential have been the subject of a detailed study.^62^

Despite the more than 40 years since its publication and the (mainly theoretical) attention that it has attracted, the article by Becker et al. contains a wealth of information that has not been sufficiently exploited and is still of current interest. Over this lapse of time, mainly integral quantities summed over final states, such as reaction probabilities, total reaction cross sections, and rate coefficients, have been reported. However, relatively few studies have been devoted to the state-to-state ICSs and DCSs, and even in those cases, the calculated DCSs or translational energy release distributions have not been used to simulate the experimental results of ref (32) in the LAB frame, which implies taking into account the spread of collision energies and the rotational state distribution of HF or HCl reagents. Moreover, the insights on the reaction mechanism discussed in the article by Becker et al. have not been contrasted with those that can be extracted from the theoretical results. The present work and its future continuation are aimed at filling this gap. In this article, we devote our attention to the simulation of the LAB ADs and TOF spectra and extract information about the correlation of the angular momenta and the distribution of collision times. In a future publication, other aspects of the reaction mechanism will be studied in detail.

The article is laid out as follows: in Section 2, the PESs used in this work are briefly described. In Section 3, the QCT methodology is discussed, with special emphasis on the calculation of the triple angle-velocity DCSs (TAV–DCS), the calculation details, the distribution of collision times, the transformation from the CM to the LAB frame, and the simulation procedure. In Section 4, the results will be presented, and some aspects will be discussed in Section 5. The conclusions will be presented in the last section.

## 2 Potential Energy Surfaces

### 2.1 Li + HF PES

The PES used for the Li + HF → LiF + H reaction in the present calculations is that by Aguado, Paniagua and Werner.^43^ Details of the ab initio calculations can be found in ref (38). The APW PES is based on ca. 6000 electronic energies, which were fitted in the many-body expansion framework with polynomials of the Rydberg functions.^63^Table 1 shows the geometries and the energies of the stationary points on this PES. The reaction from the reagent’s asymptote is endoergic by 0.117 eV (Δ_r_*H*_0_° = −0.081 eV) with a bent (∠LiFH = 72.9°) potential barrier of 0.221 eV (0.055 eV with ZPE). The PES exhibits two wells in the entrance channel. The first one is small (−0.033 eV) and corresponds to a linear approximation of the Li atom towards the hydrogen atom of the HF molecule. The deepest vdW well (−0.243 eV) corresponds to a bent approximation (104.8°) of the Li atom towards the fluorine atom of the HF molecule. The existence of the two wells is in agreement with accurate ab initio calculations^44,45^ and at variance with the most recent PES, which reports only one well.^34^ The height of the saddle point is slightly larger than the accurate ab initio result (0.221 vs 0.194 eV) but in good agreement with that given by Liu et al. of 0.215 eV.^34^ However, the APW PES and the PES of ref (34) predict only a shallow well in the exit channel of 0.067 eV (0.098 eV on ref (34)), with a bent geometry similar to that of the saddle point where the H–F distance has elongated. The ab initio calculations by Fan et al.^44,45^ predict two wells in the exit channel, the first with a geometry similar to the TS at 0.093 eV and the second one at −0.088 eV with a nearly linear geometry.

**Table 1:** Stationary Points Relative to the Reactants Asymptote in the APW^43^ PES for Li + HF → LiF + H^a^

	Li + HF → LiF + H			
	*R*(LiF)	*R*(HF)	∠LiFH	energy
reactants: Li + HF		0.917		0.0
well1: Li···H–F	4.192	0.922	0.0	–0.033
well2: Li···F–H	1.881	0.930	104.8	–0.243
saddle point	1.673	1.268	72.9	0.221
well3: Li–F···H	1.576	2.466	69.7	0.067
products: LiF + H	1.564			0.117

a Distances in Å, energies in eV, and angles in deg. ∠LiFH = 180° corresponds to the linear Li–F–H arrangement.

### 2.2 Li + HCl PES

The PES used for the Li + HCl → LiCl + H reaction in the present calculations is that by Tan et al.^60^ The TZYGL PES is built by three-dimensional cubic spline interpolation on a large number of high-level ab initio electronic energies. As shown in Table 2, this PES predicts an exoergic reaction by −0.248 eV (Δ_r_*H*_0_° = −0.0393 eV) with a bent TS (∠LiClH = 58.2°) and a height of 0.130 eV (with ZPE 0.040 eV). The PES exhibits two vdW shallow wells in the entrance channel (−0.027 and −0.018 eV) and a deeper well in the exit channel (−0.362 eV). The first entrance well corresponds to the linear Li···HCl geometry, and the second well (∠LiClH = 92.5°) corresponds to a nearly perpendicular configuration. The well located in the exit channel (∠LiClH = 52.3°) corresponds to a H···LiCl bent configuration and suggests a further twining of the hydrogen atom on the product LiCl molecule after passing the barrier and prior to flying away.

**Table 2:** Stationary Points Relative to the Reactants Asymptote in the TZYGL^60^ PES for Li + HCl → LiCl + H^a^

	Li + HCl → LiCl + H			
	*R*(LiCl)	*R*(HCl)	∠LiClH	energy
reactants: Li + HCl		1.277		0.0
well1: Li···H–Cl	4.800	1.275	0.0	–0.027
well2: Li···Cl–H	2.767	1.281	92.5	–0.018
saddle point	2.270	1.418	58.2	0.130
well3: Li–Cl···H	2.093	2.129	52.3	–0.362
products: LiCl + H	2.032			–0.248

a Distances in Å, energies in eV, and angles in deg. ∠LiClH = 180° corresponds to the linear Li–Cl–H arrangement.

The comparison of LiFH and LiClH PESs evinces a more attractive long-range interaction when the Li atom approaches the HF molecule than that with the HCl molecule. On the contrary, the exit channel is more pronounced in the case of Li + HCl. Both reactions exhibit strongly bent saddle points, with the Li–Cl–H angle being more acute.

**Figure 1:**
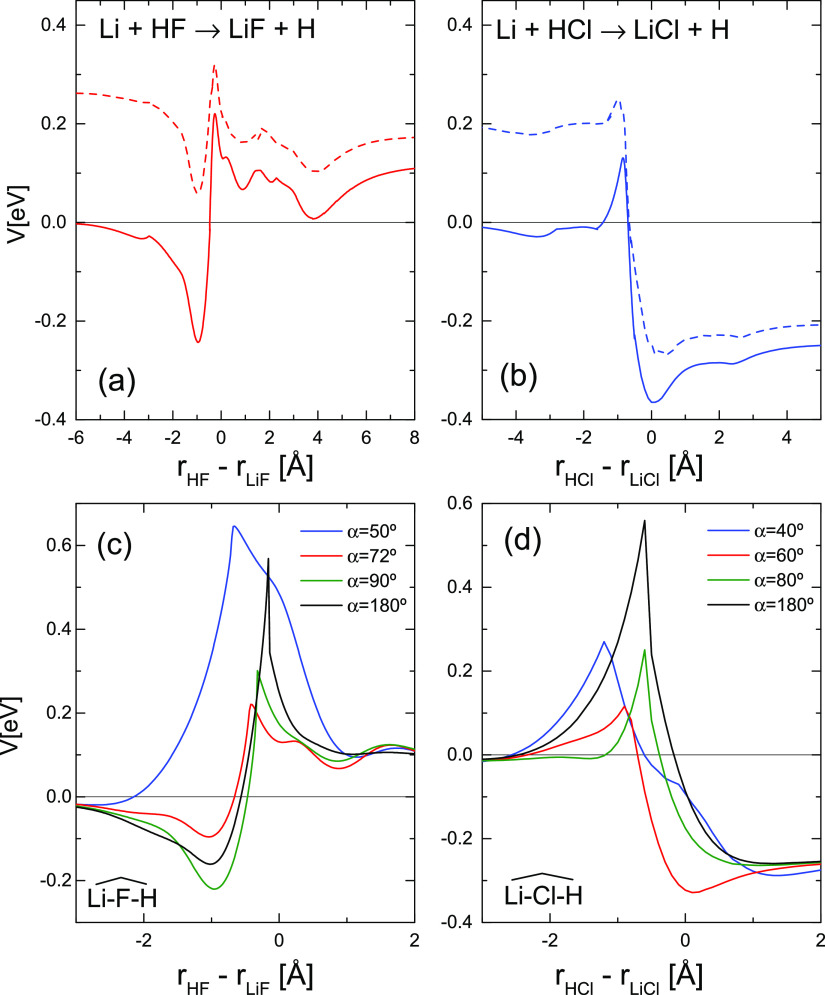
(a) MEP for the Li + HF reaction as a function of the reaction coordinate, defined as the difference between the internuclear distances *r*_HF_–*r*_LiF_, calculated by minimizing the potential over the rest of the coordinates. (b) MEP for the Li + HCl reaction. (c) and (d) Energy profiles for Li + HF and Li + HCl, respectively, as a function of the reaction coordinate at the indicated α = ∠LiXH angles. α = 180° corresponds to the linear Li–X–H arrangement.

The global minimum energy path (MEP) for the Li + HX (X = F, Cl) reactions, as a function of the reaction coordinates, *r*_HX_–*r*_XF_, are shown in panels (a) and (b) of Figure 1. Additionally, the MEPs at the indicated fixed α = ∠LiXH angles are also displayed in panels (c) and (d) of Figure 1. The presence of vdW wells at the entrance and exit channels is clearly visible, especially that of the LiFH. As can be seen, the TS barriers are quite narrow.

## 3 QCT Method

### 3.1 Calculation of the Angle-Velocity DCS as a Function of Collision Energy

It is well known that the scattering angle–recoil velocity distribution changes significantly, sometimes sharply, with both *E*_coll_ and the initial rovibrational state. Cross-molecular beam experiments, in turn, entail a spread of beam velocities—and hence of collision energies—and a distribution of reagent’s rotational states, which in cases such as those examined in this work are far from being negligible. Therefore, a reliable simulation of the raw data measured in the LAB frame must include an average over the molecular beam angular and velocity distributions and their internal state distribution. Consequently, triple scattering angle–recoil velocity DCSs, d^3^σ_R_/dω d*w*′, TAV–DCSs, must be calculated as a function of the collision energy and of the internal rovibrational states with an efficient algorithm such that it may allow us to retrieve its value every time is it needed in the simulation of the angular and TOF distributions in the LAB frame.

In previous works,^64,65^ a general method was presented to run trajectories to determine the CM TAV–DCSs as a function of the collision energy using a single batch of trajectories, followed by the simultaneous fit of the dependence of the reaction cross section on three quantities: collision energy, scattering angle, and recoil velocity. In addition, each trajectory can be sampled according to the internal state distribution. Calculations are performed by randomly and uniformly choosing the collision energy for each trajectory in a given range, [*E*_1_, *E*_2_], where the limiting energies are chosen to span the experimental distributions.

To optimize the number of trajectories leading to reaction, the dependence of the maximum impact parameter with the collision energy, *b*_max_(*E*_coll_), must be implemented. This dependence is previously determined with batches of a reduced number of trajectories run at an increasingly fixed *E*_coll_ value, until no reactive trajectory is found, and then fitting *b*_max_(*E*_coll_) with a simple functionality. Once the value of *E*_coll_ is sampled for each trajectory, *b*_max_(*E*_coll_) is calculated, and the value of *b* is sampled as *b* = ξ^1/2^*b*_max_, where ξ is a random number in the [0,1] interval. Each trajectory is then weighted with w_i_ = *b*_max_^2^(*E*_coll_)/*D*^2^, where *D* is the absolute maximum impact parameter in the whole calculation.

The procedure consists in fitting the TAV–DCSs using a Legendre moment expansion in a triple series of Legendre polynomials whose variables are the reduced collision energy, *y*, the cosine of the scattering angle, cos θ, and the reduced recoil velocity, *r*, of the detected product, all of them defined in the [−1, 1] interval and given by
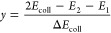
1
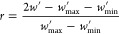
2where Δ*E*_coll_ = *E*_2_ – *E*_1_, and *w*_max_′ and *w*_min_′ are the maximum and minimum recoil velocities of the detected product in the CM frame, respectively.

The expression of the ICS, σ_R_(*E*_coll_), truncated in *k*_max_-degree is given by
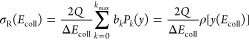
3where *P*_*k*_(*y*) is the *k*th-degree Legendre polynomial, and *Q* is the Monte Carlo estimate of the integral
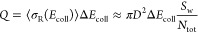
4where *N*_tot_ is the total (reactive and non-reactive) number of trajectories, and *S*_w_ is the sum of the weights of the reactive trajectories
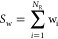
5The coefficients of the Legendre expansion, *b*_*k*_, of eq 3 are calculated as the Monte Carlo average of Legendre moments
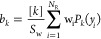
6where [*k*] ≡ (2*k* + 1)/2, and similarly for other integer numbers.

The expression of the TAV–DCS can be cast as
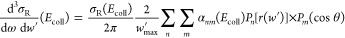
7where the collision energy-dependent coefficients, α_*nm*_(*E*_coll_), of eq 7 are given by
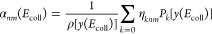
8where ρ[*y*(*E*_coll_)] is defined in eq 3. The triple-indexed coefficients, η_*knm*_, can be calculated as
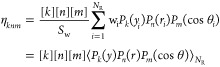
9where *y*_*i*_, *r*_*i*_, and cos θ_*i*_ are, respectively, the reduced collision energy, reduced recoil velocity, and the cosine of the scattering angle of the *i*-th trajectory. These coefficients are thus the average over the ensemble of *N*_R_ reactive trajectories of the product of the Legendre polynomials of degrees *k*, *n*, and *m* for the respective variables. The TAV–DCS can also be written as^64^
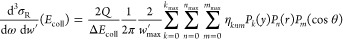
10

If the number of reactive trajectories is sufficiently large, the polar maps obtained with the triple fit are in excellent agreement with those obtained at a fixed collision energy with a θ, *r* double fit.^64^

The procedure allows us to immediately retrieve the TAV–DCS in the CM → LAB simulations. Taking into account the weighting of each trajectory, the experimental energy distribution (or beam velocity distributions), the DCS, or any other quantity (as the opacity function) can be determined by averaging over the energy and internal state distributions.

### 3.2 Distribution of Collision times

When analyzing reaction mechanisms, it is important to determine the collision time, τ_coll_, roughly defined as the lapse of time that the atoms spend together forming a complex before it breaks down to form products or reagents. Usually, collision times furnish valuable information about the nature of the collision mechanisms, which sometimes coexist and lead to different τ_coll_ distributions.^66−68^ For atom-diatom reactions, collision times span a very wide range of times, from a few femtoseconds to tens of picoseconds, depending on the features of the PES and the collision energy. Moreover, specific outcomes of a collision (rovibrational state, scattering angle, etc.) are generally associated with different τ_coll_, such that it is possible to determine the time evolution of scattering. Nevertheless, a precise definition of collision time is not exempt from debate. The most reliable criterion is to rely on the behavior of the potential. When the three atoms involved in an atom-diatom collision are close to each other, the potential is strongly perturbed with respect to its asymptotic behavior. This is illustrated in the Supporting Information Figure S1, where the three internuclear distances and the potential energy are represented as a function of time for the Li + HF (*v* = 0, *j* = 0) reaction at *E*_coll_ = 0.378 eV. However, detecting when the three-body contribution to the potential becomes important is not trivial. Fortunately, examination of the trajectories as a function of time for a given reaction shows that this generally occurs when the initial and final distances from the atom to the diatom CM, *R* and *R*′, become smaller than *R*_int_ and *R*_int_^′^, respectively. Therefore, *R*_int_ and *R*_int_^′^ serve to define the strong interaction region, at least at not too low energies. The values of these limiting distances depend on the reaction and are determined by following the time evolution of the interatomic distances and potential energy for a significant number of trajectories. Usually, *R*_int_ and *R*_int_^′^ are within 2–3 Å. Once the values have been defined, the collision time for a given trajectory can be calculated as^67,68^
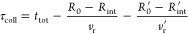
11where *t*_tot_ is the total duration of the trajectory, *R*_0_ and *R*_0_′ are the initial and final atom-diatom cm distances, respectively, and *v*_r_ and *v*_r_′ are, respectively, the asymptotic initial and final relative velocities. Accordingly, the collision time would be the time delay between the beginning of the interaction, when the reagents meet at a distance *R*_cm_ = *R*_int_, and the formation of products, when *R*_cm_^′^ = *R*_int_^′^. In a hypothetical trajectory in which the formation of the products takes place instantaneously, its collision time would be equal to zero; a real trajectory, even the most direct one, will have a finite collision time, implying that there is a delay between the beginning of the interaction and the formation of products. The advantage of using eq 11 is that there is no need to recalculate reactive trajectories to determine their collision time. A more accurate method, based on monitoring the changes in the potential in search of the onset of the three-body interaction, shows that the error in estimating τ_coll_ for a given trajectory is very small.

The distribution of collision time, *P*(τ_coll_), can be carried out using histograms or by fitting the results to a series of Legendre polynomials and can also be determined in conjunction with other observables, such as scattering angle, internal state of the products, or impact parameter. It is also interesting, as shown in previous works,^67,68^ to plot the joint probability distribution of scattering angles and collision times, *P*(τ_coll_, θ), rendering information not only on when the products are formed but also on how they are scattered in space. The calculation of *P*(τ_coll_, θ) can be carried out as a double expansion in Legendre polynomials or, alternatively, in a series of Gaussian functions given by
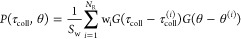
12where *N*_R_ is the number of reactive trajectories, *w*_*i*_ is the weight of the *i*-th trajectory, and *S*_w_ is the sum of the weights. τ_coll_^(*i*)^ and θ^(*i*)^ are the values of the collision time and the scattering angle of the *i*-th trajectory, respectively. *G*(τ_coll_ – τ_coll_^(*i*)^) and *G*(θ – θ^(*i*)^) denote normalized Gaussian functions centered in τ_coll_^(*i*)^ and θ^(*i*)^, with width parameters δ_τ_ and δ_θ_, respectively. The sum runs over the whole ensemble of trajectories at a given collision energy. Both methods render very similar results with essentially the same computational effort.

### 3.3 Calculations Details

As mentioned in Section 2, calculations for the Li + HF and Li + HCl reactions were carried out on the APW PES and the TZYGL PES, respectively. The APW PES was fitted with a very fast analytical expression that includes the derivatives. The TZYGL PES is based on a spline fit, and the derivatives are not analytical. QCT calculations on this PES are about 50 times slower than on the APW PES.

For the Li + HF reaction, a batch of 25 × 10^6^ trajectories was run in the 0.01–1.00 eV interval for a distribution of initial rotational states corresponding to a rotational temperature of 120 K, as estimated from the nozzle stagnation temperature and the experimental translational temperature. Since the maximum impact parameter at each collision energy, *b*_max_(*E*_coll_), decreases (for Li + HF) or increases (for Li + HCl) with *E*_coll_, the impact parameter *b* for each trajectory was sampled by taking into account the dependence of *b*_max_ on *E*_coll_, as indicated above. For Li + HF, *b*_max_ changes from 4 Å at 0.01 eV to 1.9 Å at *E*_coll_ > 0.3 eV. The absolute maximum value of the impact parameter is *D* = 5 Å. For Li + HCl, a batch of 17 × 10^6^ uniformly distributed trajectories in the 0.01–1.00 eV interval was run on the TZYGL PES. *b*_max_(*E*_coll_) first increases rapidly with *E*_coll_ and then stabilizes. The *D* parameter was set to 2.7 Å. Additional batches of 2–5 millions of trajectories at fixed collision energies and specific rotational states were calculated to study some aspects of the reaction dynamics, such as vector correlations or the distribution of collision times. Trajectories were started and completed at an atom-diatom distance of 10 Å, and the integration step size was chosen to be 0.05 fs, which guarantees better energy conservation than 1 part in 10^5^.

The rovibrational energies of HF, HCl, LiF, and LiCl were calculated using the Numerov algorithm with the respective asymptotic diatomic potential of each PES. The assignment of product final quantum numbers was done as in previous works by equating the classical internal energies to Dunham series expansions, whose coefficients have been previously determined by fitting the QM exact rovibrational energies. The resulting classical (real) vibrational, *v*′, and rotational, *j*′, values were rounded to the nearest integers. Since the vibrational and rotational quanta of the LiF and LiCl are relatively small, there is no need to apply procedures such as Gaussian binning.

To check the validity of the QCT calculations, we calculated the cumulative reaction probabilities (CRPs) for total angular momentum *J* = 0 using both QCT and QM calculations. The latter were calculated using the ABC code.^69^ Once the *S*-matrix is extracted from the QM calculations, the CRP is calculated as
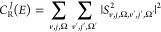
13where the sum runs over all rovibrational states of reactants and products and all values of the helicity (projection of *J*, *j*, or *j*′ onto the initial or final relative velocities). *E* denotes the total (collision plus rovibrational) energy.

Details of the calculations of QCT CRPs can be found in ref (70). The expression of the CRP at a given total energy, *E*, and total angular momentum, *J*, is
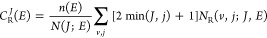
14where *N*_R_(*v*, *j*; *J*, *E*) is the number of reactive trajectories, (*v*, *j*) is the initial rovibrational state, *N*(*J*; *E*) is the total number of trajectories ran at a given *E* and *J*, and *n*(*E*) is the number of open reactant states at a given energy.

To calculate the QM CRP for Li + HF, the maximum energy was *E*_max_ = 1.8 eV, *j*_max_ = 200 and a maximum hyperradius ρ_max_ = 23 a_0_ and 345 sectors. The respective parameters in the calculation for Li + HCl have been *E*_max_ = 2.8 eV, *j*_max_ = 400, ρ_max_ = 50 a_0_ and 4000 sectors.

### 3.4 Simulation of the Experimental Results

The simulation of the LAB AD of scattered LiF and LiCl molecules has been performed by transforming the theoretical CM TAV–DCSs into the LAB system.^38,39,71^ The signal at a LAB angle Θ_LAB_ can be expressed as
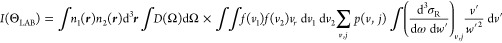
15where *n*_1_(***r***) and *n*_2_(***r***) are the (relative) spatial beam densities, *D*(Ω) function accounts for the detector aperture, *f*(*v*_1_) and *f*(*v*_2_) are the reagent beam velocity distributions, and *p*(*v*, *j*) is the distribution of rovibrational energies in the HF or HCl beam, which usually is taken as a Boltzmann distribution at the rotational temperature *T*_rot_. *v*_*r*_ is the reagent relative velocity, and *v*′/*w*′^2^ is the Jacobian of the CM to LAB transformation for product density detection, where *v*′ and *w*′ are the LAB and CM product velocities, respectively. Since the batch of trajectories was run by sampling the initial rotational distribution and the calculation is rendering *w*′ for each trajectory, the calculated TAV–DCS already includes the averaging over the internal state distribution.

The TOF spectra were simulated as in previous works,^38,64,71,72^ and the necessary experimental parameters for the simulation were taken from ref (32). The signal at a given time *t* and LAB angle Θ_LAB_ can be expressed as
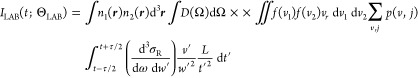
16where *L* is the flight-path length, *t* is the corresponding time of flight of the formed product, and the factor *L*/*t*′^2^ accounts for the transformation from velocity to time space. The experimental *L* was set at 17 cm, and the channel width, τ, was taken to be 12 μs, as given in ref (32).

## 4 Results

**Figure 2:**
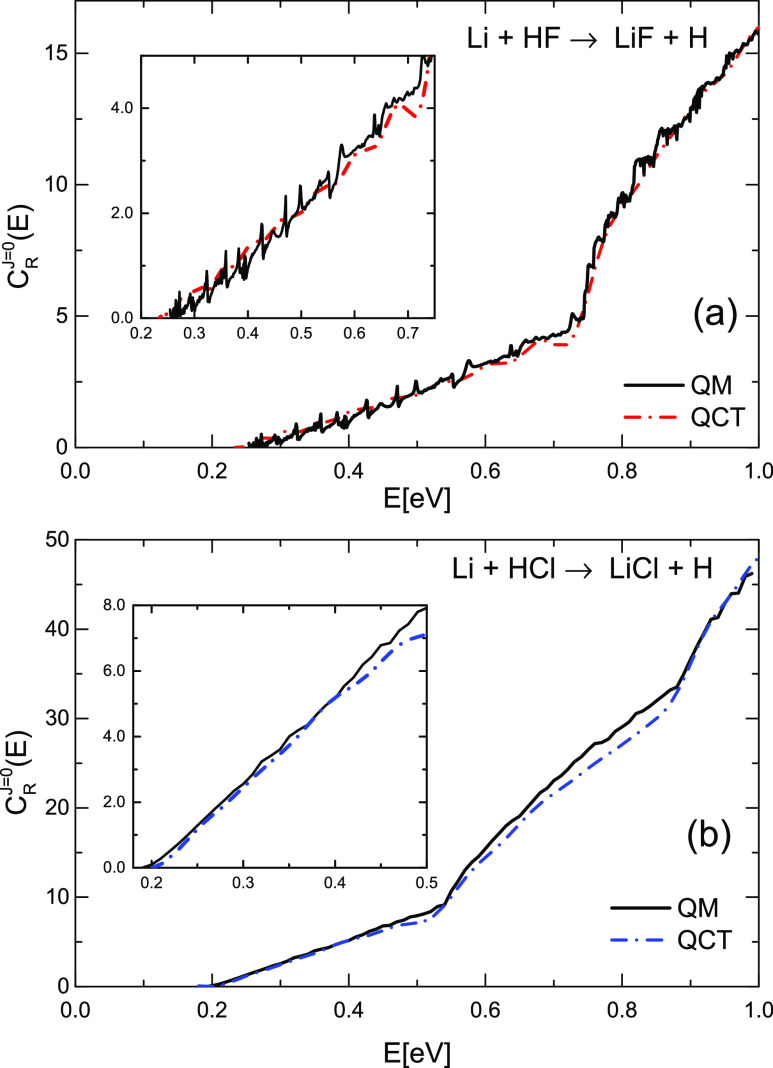
Comparison of QM (solid lines) and QCT (dot dashed lines) CRPs for *J* = 0 as a function of the total energy for the Li + HF (a) and Li + HCl (b) reactions.

Figure 2 shows the comparison between the QM and QCT CRPs for the two reactions as a function of the total energy and for zero total angular momentum, *J*. Apart from the fine-grained structure, which is apparent in the QM CRP for the Li + HF reaction, indicating the presence of resonances, the agreement is very good. The change in slope at 0.78 eV corresponds to the opening of the HF *v* = 1 manifold. Some steps and peaks can be observed, but it is difficult to discern whether they are due to the opening of LiF channels or to resonances. The comparison for Li + HCl (panel (b)) also shows good agreement, although there are some deviations after the opening of the HCl *v* = 1. The CRP is a quantity summed over all internal states of reactants and products, and its intrinsic dynamical significance is limited. However, the agreement between QCT and QM CRPs indicates that the former are reliable as a faithful approximation to their QM counterparts.

**Figure 3:**
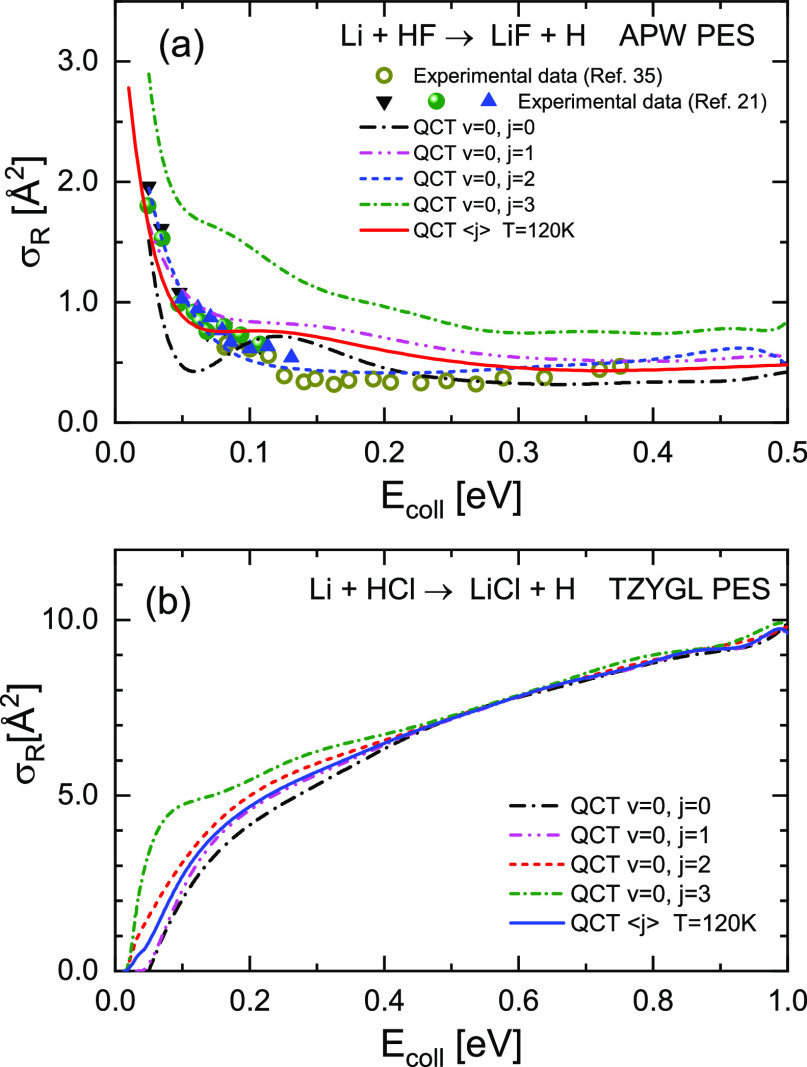
QCT reaction cross sections for (a) Li + HF → LiF + H and (b) Li + HCl → LiCl + H reactions as a function of the collision energy (excitation function) for the indicated initial rotational states. The excitation functions averaged over the rotational state distribution at *T*_rot_ = 120 K are also shown as solid lines for both reactions. The Li + HF excitation functions are compared with the experimental data from refs (21) and (35), for which only relative values were determined. For comparison purposes, the experimental data have been scaled to the theoretical results by least squares. Note the remarkable difference in the shape and scale of the excitation functions for the two reactions.

The excitation functions, σ_R_(*E*_coll_), for the Li + HF (a) and Li + HCl (b) reactions and for different initial rotational states are shown in Figure 3. In spite of a similar kinematics for the two reactions, H + HL → HH + L, they exhibit a very distinct behavior in their respective dynamical observables and, in particular, in their σ_R_(*E*_coll_). For Li + HCl, QCT calculations predict a threshold at a low collision energy (<0.05 eV) for all the rotational states, followed by a monotonic rise of σ_R_(*E*_coll_) up to *E*_coll_ = 1.00 eV. In contrast, calculations for the Li + HF predict no reaction threshold and a quick decrease with *E*_coll_ until ≈0.1 eV when σ_R_(*E*_coll_) levels off. Moreover, the absolute values of σ_R_(*E*_coll_) are very different for the two reactions, as can be seen in Figure 3. In the case of the Li + HF reaction, its value is 3 Å^2^ at *E*_coll_ ≈ 0.01 eV, but at energies above 0.05 eV, σ_R_(*E*_coll_) becomes 0.5–1.0 Å^2^, depending on the HF rotational state. For the Li + HCl, at *E*_coll_ > 0.3 eV the reaction cross sections grows from 5 Å^2^ at 0.3 eV to 10 Å^2^ at 1.0 eV.

Figure 3a also shows the comparison between the experimental data by Loesch and co-workers,^21,35^ covering the range 0.025–0.376 eV. Of particular interest are the experimental results at *E*_coll_ ≤ 0.082 eV,^21^ because previous QM calculations on various PESs predicted an effective threshold or a decrease in σ_R_(*E*_coll_) below this collision energy.^53,55,73−75^ Only relative values of the reaction cross section were measured in these experiments, and for a proper comparison, the experimental data have been scaled by least squares to the present QCT results on an absolute scale. In general terms, the agreement between the two sets of results is satisfactory, especially when the excitation function for *j* = 2 is compared with that scaled to the experimental data. In the experiment, the average rotational state in the HF beam was estimated to be ⟨*j*⟩ = 1.4, which roughly corresponds to a Boltzmann rotational temperature of 140 K.^21^

The present results can also be compared with the estimation of the absolute value by Becker et al.^32^ based on the comparison of reactive scattering signals with small-angle elastic signals, and using the theoretical small-angle elastic signal calculated with the vdW long-range interaction. An estimate of the rotational temperature in the experiments of Becker et al., based on the translational temperature of the HF beam and the nozzle stagnation temperature, is 120 K. With this rotational state distribution, which results in ⟨*j*⟩ = 1.25, the cross section values at *E*_coll_ = 0.132 eV (corresponding to an experimental nominal energy of 3.0 kcal mol^–1^) and at *E*_coll_ = 0.393 eV (experimental nominal energy 8.7 kcal mol^–1^) are 0.78 and 0.53 Å^2^, respectively, to be compared with 0.80 and 0.94 Å^2^ deduced in the mentioned work. Considering the inherent uncertainties, these estimations are in very good agreement with the QCT results. The QCT excitation function averaged over the HF rotational distribution is shown in Figure 3, in an acceptable agreement with the experimental data.

Hazra and Balakrishnan carried out extensive, accurate TI-QM calculations at low collision energies for the Li + HF (*v* = 0, *j* = 0) on the AP2 PES,^76^ as well as on the APW PES used in this work.^33^ Whereas the excitation function on the AP2 PES displays a sharp decrease below *E*_coll_ = 10^–3^ eV and an “effective” threshold (i.e., when σ_R_ < 5 × 10^–5^ Å^2^), calculations on the APW PES, predict a monotonic increase in σ_R_(*E*_coll_) up to *E*_coll_ = 10^–5^ eV, when it levels off but without any trace of an effective threshold. Apart from fairly sharp oscillations due to resonances in the QM results, the QCT calculations offer a good approximation to the QM results with absolute values in good agreement. The difference between the results on the AP2 and APW PESs has been attributed to the higher barrier in the former PES (0.233 eV vs. 0.221 eV on the APW PES). Calculations on the APW were also carried out by Zanchet et al. using wave packet TD-QM method.^55^ However, a threshold was found at ≈ 0.01 eV in contrast to the results obtained with TI-QM by Hazra and Balakrishnan.^33^ TD-WP calculations by González-Sánchez et al. were also performed on the APW PES.^56^ The resulting σ_R_(*E*_coll_; *j*) with *j* = 0–3 is in good agreement with the present results, although the rise below 0.05 eV is not as sharp as in the QCT calculation. QTD-WP calculations for the Li + HF (*v* = 0, *j* = 0) reaction have also been carried out on the PES by Liu et al.^34^ The resulting excitation function is similar to that obtained on the APW PES, although the absolute values of σ_R_ are slightly larger throughout the whole interval of *E*_coll_. No threshold was found, but in contrast to the QCT results, only for *E*_coll_ < 0.02 eV did the cross section increase abruptly.

As commented on in Section 1, the values of σ_R_ derived by Becker et al. for the Li + HCl reaction at *E*_coll_ = 0.132 eV and *E*_coll_ = 0.399 eV were 27 and 54 Å^2^, respectively. They are a factor of 6 larger than those found by the present QCT calculations, which are in good agreement with the QM results by Tan et al. on the same TZYGL PES.^60^ The main difference between the present QCT and the QM calculations is that the QM threshold is practically zero and disappears for *j* = 1 and 2, whereas the QCT calculations predict thresholds at *E*_coll_ ≤ 0.05 eV. Similar results were obtained by Zhai et al. for *j* = 0 on the same PES using CC–TD calculations.^77^

**Figure 4:**
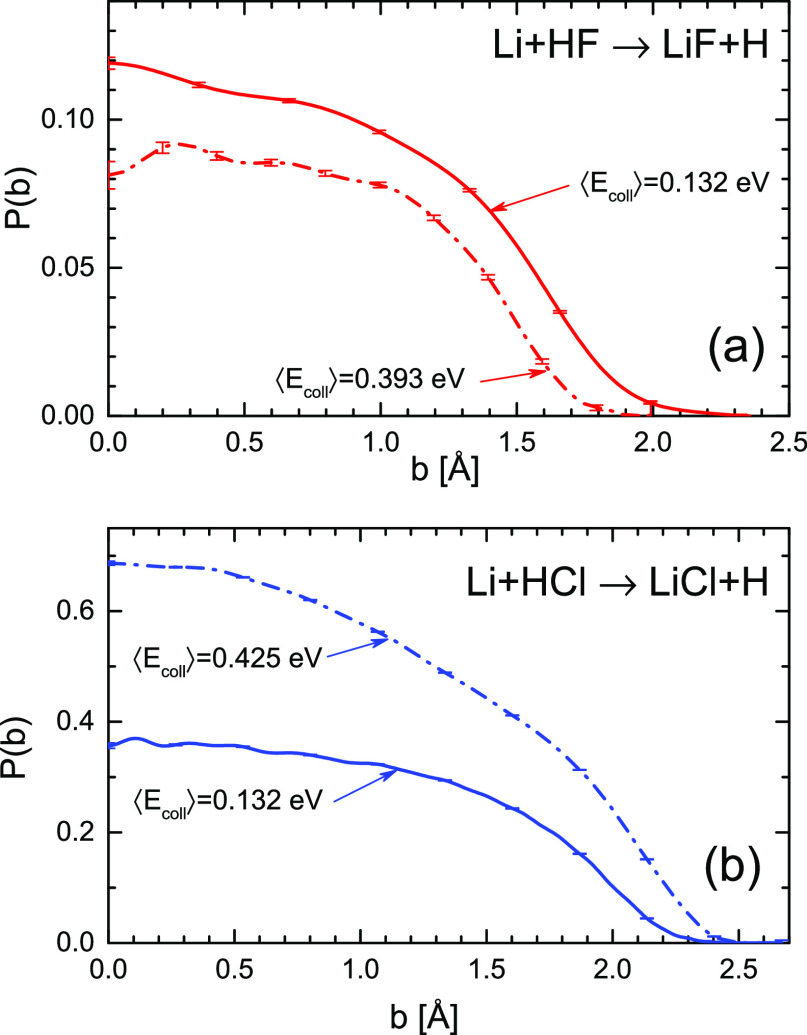
Reaction probability as a function of the impact parameter (opacity functions) for Li + HF → LiF + H (a) at the mean collision energies of 0.132 and 0.393 eV, and Li + HCl → LiCl + H (b) at 0.132 and 0.425 eV. The respective opacity functions have been averaged over the experimental translational and rotational reactant’s energy distributions. The opacity function for Li + HF is considerably smaller for the higher collision energy, so is the maximum impact parameter. In contrast, the opacity function for Li + HCl, whose values are much bigger, grows with collision energy.

Figure 4 displays the opacity functions (reaction probabilities as a function of the impact parameter), *P*(*b*), at the mean collision energies of 0.132 eV (nominal energy 3 kcal mol^–1^) and 0.393 eV (nominal energy 8.7 kcal mol^–1^) for the Li + HF, and at the mean collision energies of 0.132 eV (nominal energy 2.9 kcal mol^–1^) and 0.425 eV (nominal energy 9.2 kcal mol^–1^) for the Li + HCl. The opacity functions have been calculated by averaging the QCT results over the experimental collision energy and initial rotational distributions. As can be seen, the behavior of the respective *P*(*b*) is very different. For Li + HF, the values of the probability are much lower than in the case of the Li + HCl reaction, in accordance with the much higher cross-section values for the latter. Besides, with increasing *E*_coll_, the *P*(*b*) becomes lower, and so does the maximum impact parameter for Li + HF. The opposite behavior takes place in the Li + HCl reaction. While the behavior of *P*(*b*) with *E*_coll_ in the former is reminiscent of a harpooning mechanism or a statistical reaction, the *P*(*b*) of the latter reaction corresponds to a direct reaction with a barrier.

**Figure 5:**
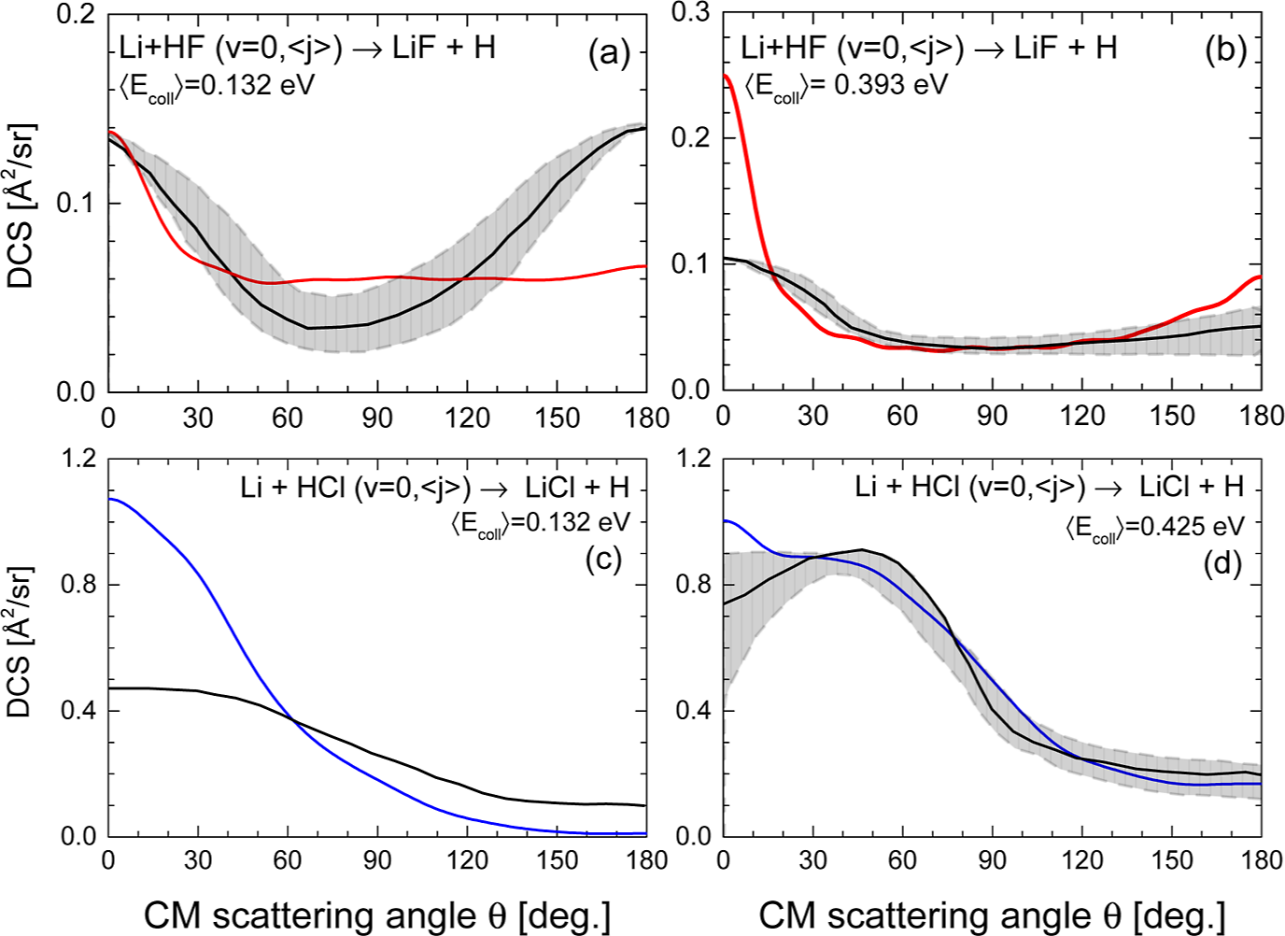
Comparison of the experimentally derived (black solid line) DCSs (ref (32)) and the corresponding QCT DCSs (red solid line for the Li + HF reaction and blue line for the Li + HCl reaction), obtained by averaging the results over the velocity and rotational distributions of the molecular beams. Top panels: Li + HF reaction at the average collision energy, ⟨*E*_coll_⟩, of 0.132 eV (a) and 0.393 eV (b) (3.0 and 8.7 kcal mol^–1^ nominal energies). Bottom panels: Li + HCl reaction at ⟨*E*_coll_⟩ = 0.132 eV (c) and 0.425 eV (d) (2.9 and 9.2 kcal mol^–1^ nominal energies). The experimental DCSs were derived by fitting the measured LAB AD and TOF distributions to scattering angle–recoil velocity distributions assumed to be uncoupled.^32^ The uncertainty in the derivation of the experimental DCS is shown as a shaded region.

In Figure 5, the experimentally derived and the QCT DCSs are compared for the two reactions. The QCT DCSs have been calculated by averaging over the collision energy and rotational state distributions of the reactants.^32^ In turn, the experimental CM DCSs were obtained in ref (32) by forward convolution trial and error to reproduce the measured LAB ADs and TOF spectra, assuming separable (independent) angular and velocity distributions in the CM frame. For Li + HF, the agreement between the experimental and theoretical DCSs is satisfactory at the highest energy, but they clearly differ at the lower one. At ⟨*E*_coll_⟩ = 0.132 eV, the present QCT calculations on the APW PES predict a predominantly flat, isotropic DCS, except in the forward region, θ ≤ 30°, in contrast with the nearly backward–forward symmetric angular distribution derived from the experiment. For the Li + HCl reaction, QCT calculations predict a clear forward angular distribution, while the experimentally derived DCS is broader with a non-negligible backward component.

Liu et al.,^34^ using QM TD wave packets, determined the DCSs using the PES for Li + HF by these authors. The shapes of the DCSs for *j* = 0 are in fairly good agreement with those of the present QCT DCSs and are indeed closer to the present results than those derived experimentally. Similar results were obtained in ref (56) for various initial *j*’s on the APW PES. It must be taken into account that the DCSs in both references correspond to the nominal collision energies without averaging over the collision energy or the rotational state distributions.

**Figure 6:**
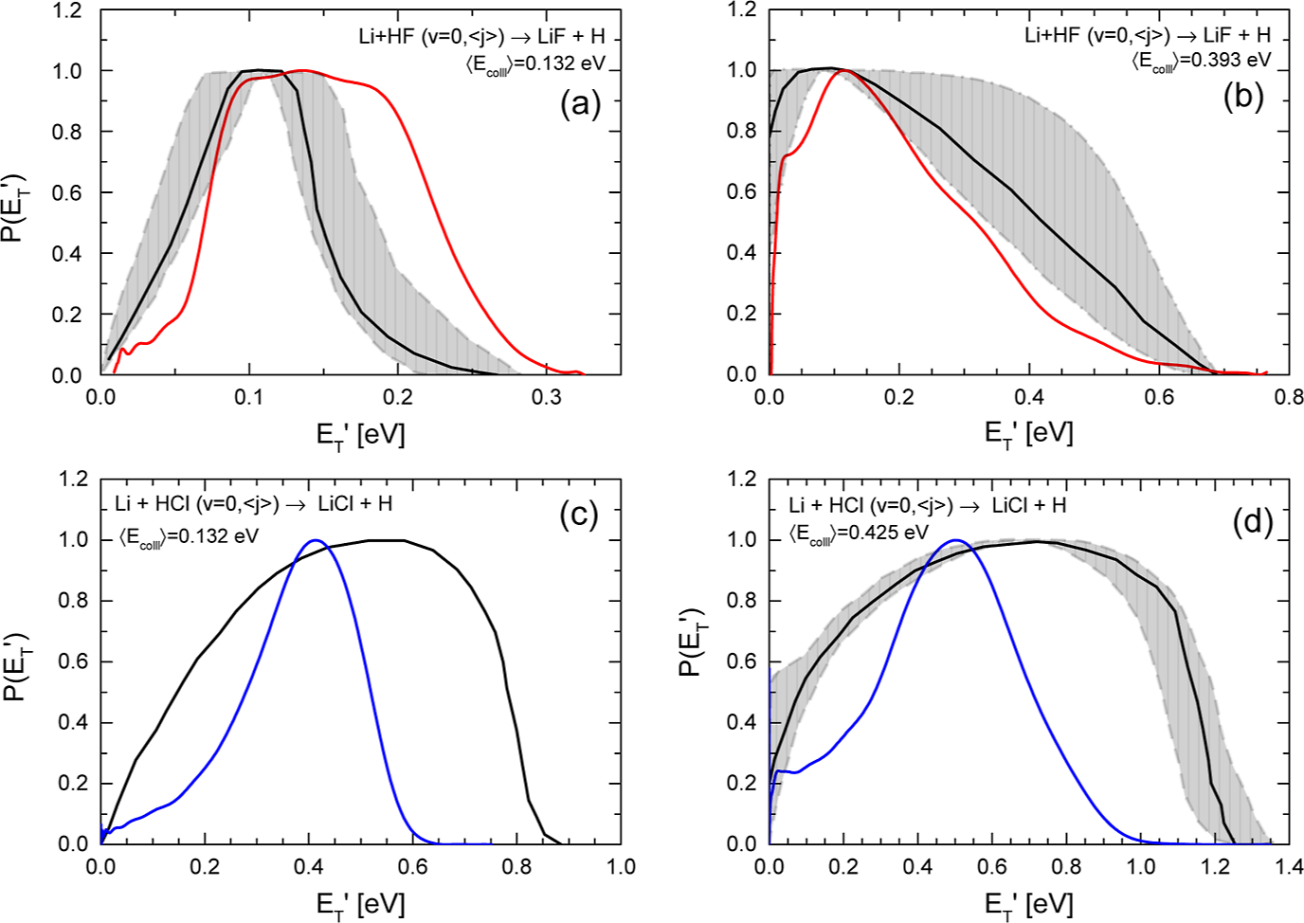
Comparison of the experimentally derived (black lines and error bars indicated by the shaded regions) product’s energy distributions, *P*(*E*_T_′), from ref (32) with those obtained in the present QCT calculations for Li + HF (red lines) and Li + HCl (blue lines) at the indicated average collision energies, that correspond to the nominal values of 3.0 kcal mol^–1^ (a) and 8.7 kcal mol^–1^ (b) for Li + HF, and 2.9 kcal mol^–1^ (c) and 9.2 kcal mol^–1^ (d) for Li + HCl.^32^ The QCT results have been averaged over the velocity and rotational distributions of the molecular beams. The experimental recoil energy distributions have been derived assuming uncoupled angle-velocity distributions.

The comparison between the experimentally derived recoil energy distributions, *P*(*E*_T_′), and those obtained in the present calculations is shown in Figure 6. As in the case of the DCSs, the theoretical results have been averaged over the experimental beam velocity and rotational energy distributions of the collision partners. The QCT *P*(*E*_T_′)’s for Li + HF are closer to the experimentally derived results, although for ⟨*E*_coll_⟩ = 0.132 eV they are substantially broader. For the Li + HCl reaction, the theoretical distributions are much narrower than the experimental ones, and the maximum recoil energy is clearly lower. The width of the experimentally derived *P*(*E*_T_′) has not been reproduced by any of the previous calculations^57,58^ and cannot be explained by the averaging over the experimental collision energy distribution, which, as will be seen, has a noticeable influence on the width of the LAB AD.

The present QCT average fraction of the available energy into translational energy, ⟨*f*_T_⟩, is 56% for Li + HF at ⟨*E*_coll_⟩ = 0.132 eV, while the respective fractions on vibration and rotation are 18 and 26%, respectively. Based on their *P*(*E*_T_′), Becker et al. concluded that ⟨*f*_T_⟩ = 55% with the remaining available energy predominantly channeled into rotation, which is in very good agreement with our present calculations. At ⟨*E*_coll_⟩ = 0.393 eV, the calculated fractions of the total energy into translation, vibration, and rotation are 38.5, 33.1, and 28.4%, respectively. Becker et al. guessed an increase of ⟨*f*_v_⟩ at the expense of the rotational temperature, in contrast to the trajectory results available at that time.

The ⟨*f*_T_⟩, ⟨*f*_v_⟩ and ⟨*f*_R_⟩ values for the Li + HCl reaction obtained in the present calculations at ⟨*E*_coll_⟩ = 0.132 eV, are 64.4, 24.3, and 11.3%, respectively. At the highest collision energy, ⟨*E*_coll_⟩ = 0.435 eV, the values of *f*_T_, *f*_v_, and *f*_R_ are 55.1, 21.3, and 23.6%, respectively. The value of ⟨*f*_T_⟩ inferred by Becker et al. was 70% for both collision energies.

**Figure 7:**
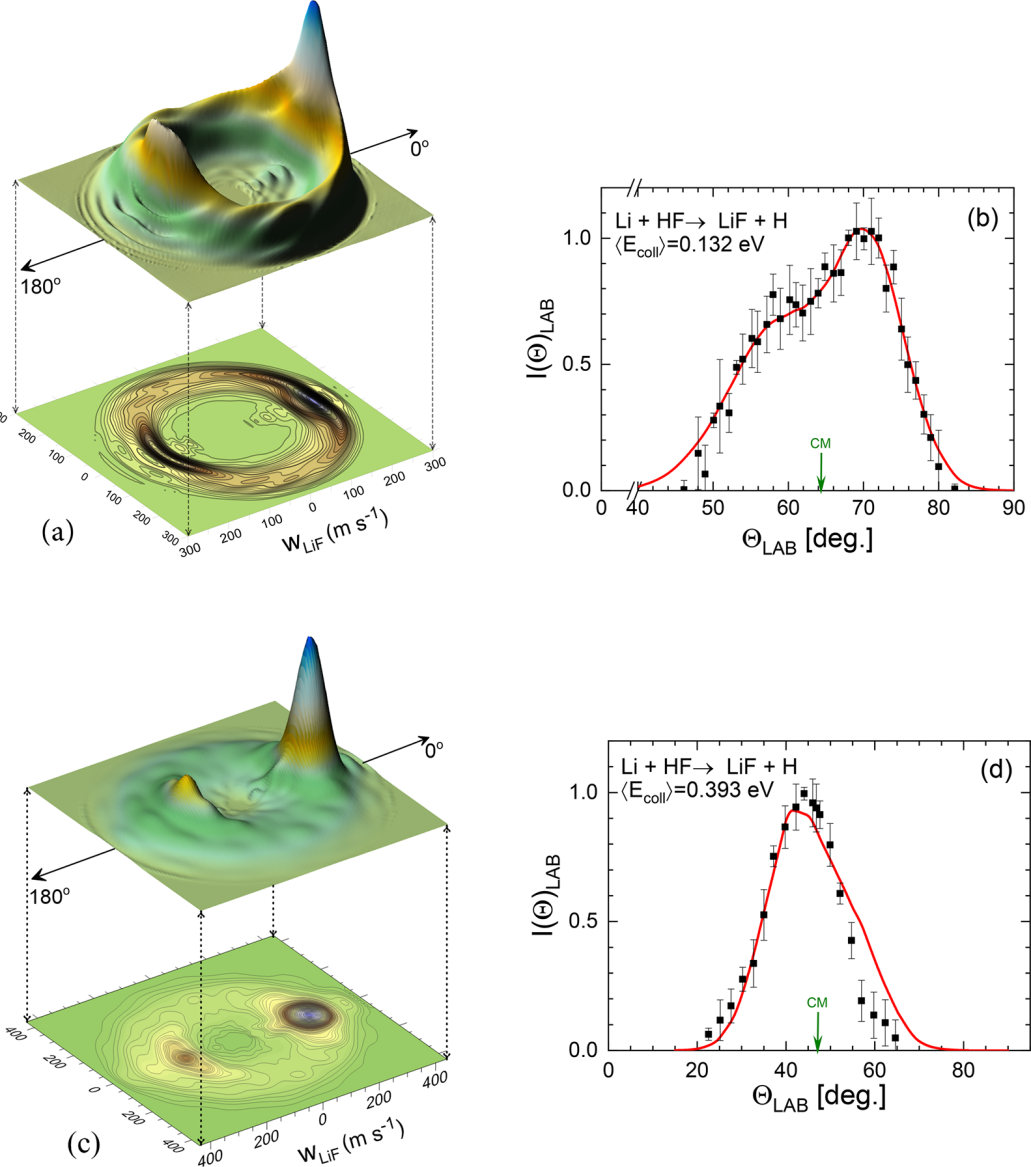
Left panels: three dimensional perspectives and contour polar plots of the TAV–DCS for the Li + HF reaction at ⟨*E*_coll_⟩ = 0.132 eV (a) and ⟨*E*_coll_⟩ = 0.393 eV (c). (b, d): comparison between the experimental (solid squares with error bars) from ref (32) and the simulated LiF LAB AD (red line) using the present theoretical TAV–DCS as a function of the collision energy, taking into account the experimental velocity distributions of the Li and HF beams and the rotational state distribution of HF assuming a rotational temperature of 120 K.

The angle-velocity polar maps have been calculated by averaging over the collision energy distribution and the internal state distributions of HF or HCl, as described in Section 3. Those simulated for Li + HF at ⟨*E*_coll_⟩ = 0.132 eV and ⟨*E*_coll_⟩ = 0.393 eV are shown in panels (a) and (c) of Figure 7. Although both polar maps bear obvious resemblances to those obtained by forward convolution by trial and error in ref (32), there are clear differences. The source of the discrepancy lies in the derivation of the latter, which assumes that the CM distributions were separable, that is,
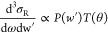
17where, in addition, the dependence of σ_R_(*E*_coll_) within the experimental collision energy distribution was neglected.

A careful comparison of the contour plots of ref (32) and those calculated in this work reveals some differences. At the lowest *E*_coll_, there is a clear predominance for forward scattering, and although there is a peak in the backward region, it is considerably smaller and confined at intermediate velocities. Clearly, the angular distribution depends on the product velocity (that is, the LiF internal state). Similarly, at ⟨*E*_coll_⟩ = 0.393 eV, the velocity distribution in the forward region differs from that at backward angles. Given the differences between the theoretical and experimentally derived DCS and recoil velocity distributions, the issue is whether the present calculations are able to account for the raw data in the LAB frame.

To check the differences that would result in our polar maps assuming the separability of the angular and velocity distributions, we have calculated the TAV DCS as the product of the *P*(*w*′) and the DCS. This is equivalent to making the coefficients of eq 7, α_*mn*_ ∝ α_*m*0_·α_0*n*_. The corresponding polar maps are displayed in Figure S2 of the Supporting Information, where the differences with those portrayed in Figure 7 are evident.

The LAB ADs simulated for both collision energies with the present QCT calculations using the procedure summarized in eq 15 are plotted in the right panels of Figure 7, together with the experimental data from ref (32). It must be emphasized that no adjustable parameters were used and that the simulations were carried out with the TAV DCS as a function of the collision energy and the HF internal state. The agreement is excellent at ⟨*E*_coll_⟩ = 0.132 eV and acceptable at ⟨*E*_coll_⟩ = 0.393 eV, where the simulated LAB AD is slightly wider at LAB angles above those corresponding to the center of the mass.

**Figure 8:**
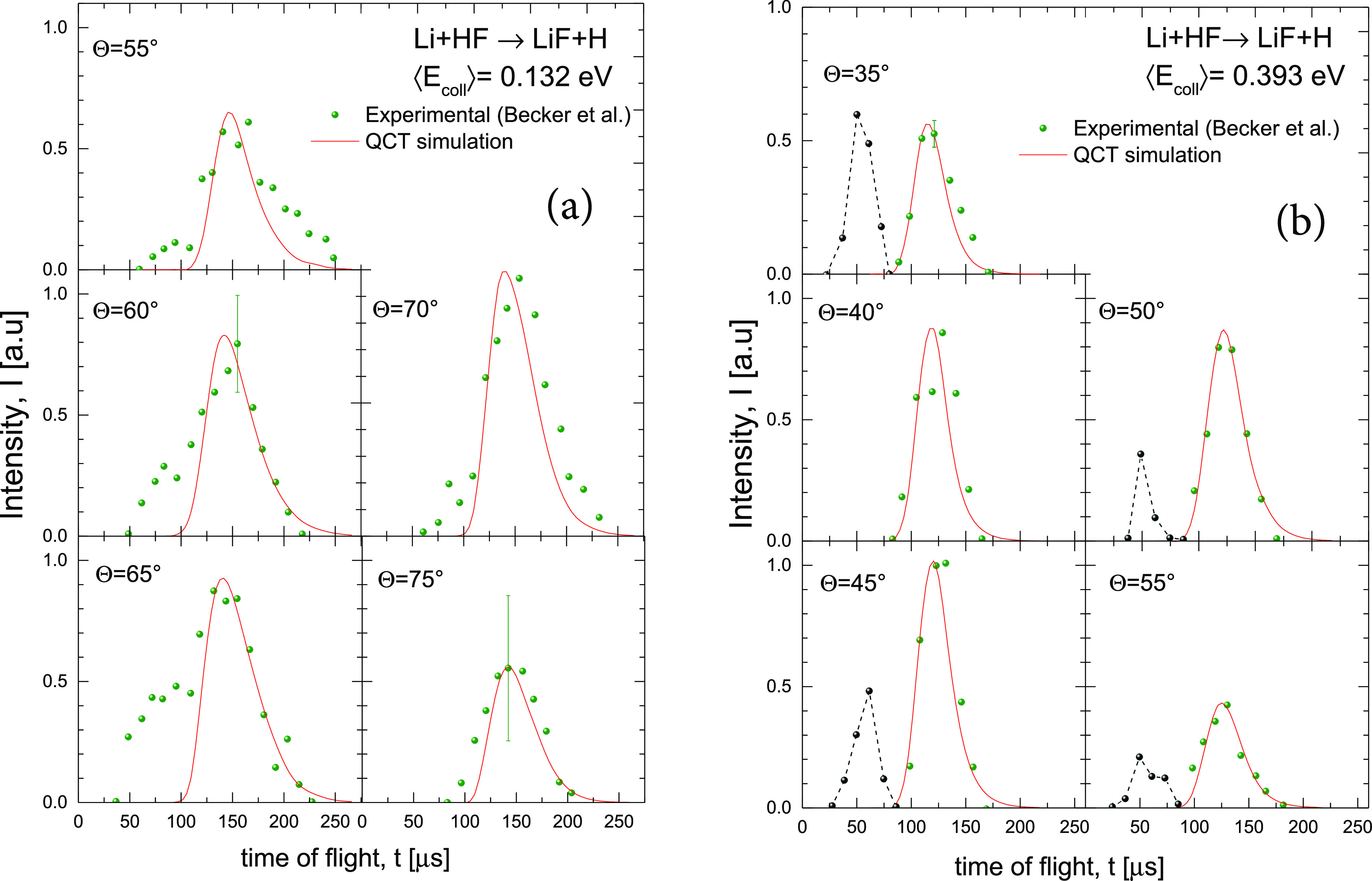
Comparison of experimental^32^ (solid symbols) and simulated (red line) TOF distributions of the LiF product at ⟨*E*_coll_⟩ = 0.132 eV (a) and at ⟨*E*_coll_⟩ = 0.393 eV (b) using the present theoretical results at the indicated LAB angles. The TOFs shown in the figure correspond to the nominal energies *E*_coll_ = 3.0 kcal mol^–1^ and *E*_coll_ = 8.7 kcal mol^–1^, shown in ref (32). Black dash lines and points represent the experimental non-reactive scattering.

The comparison of the simulated TOF spectra and the experimental points for Li + HF at the two collision energies is shown in Figure 8. The agreement is very good, at least as good as the one obtained with the fit of ref (32). The dashed lines drawn in the simulations for ⟨*E*_coll_⟩ = 0.393 eV are due to non-reactive scattered Li since the TOF spectra were measured with *m*/*z* = 7. The shoulders corresponding to fast scattering (low LAB angles) for ⟨*E*_coll_⟩ = 0.132 eV have the same origin. These results confirm the validity of the QCT calculations on the APW PES, which was already shown in the good agreement between simulated and experimental LAB results for this reaction,^38^ and the need to take into account the coupling between velocity and angular distributions.

**Figure 9:**
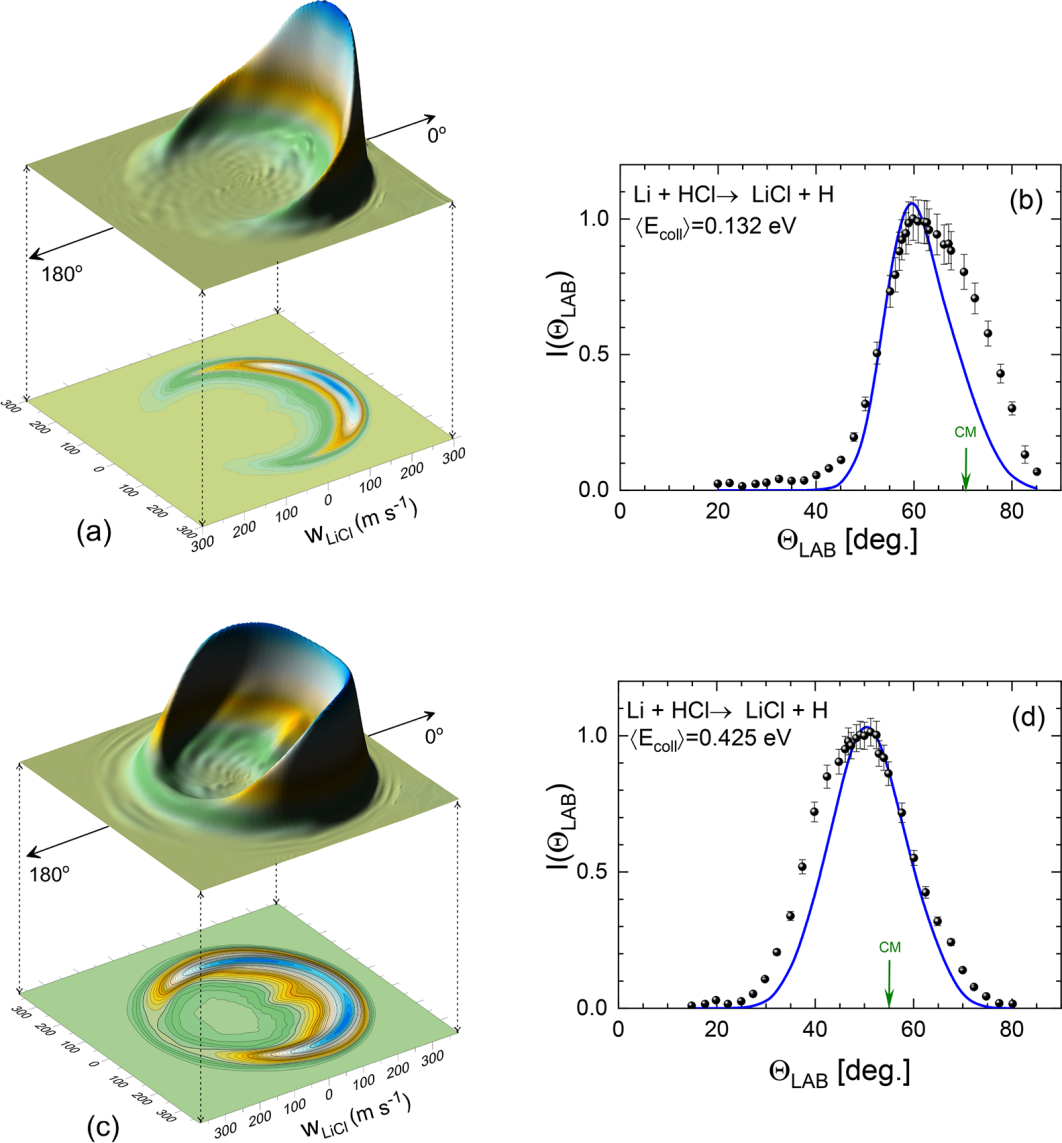
Three dimensional perspective and contour polar plots of the TAV DCSs for the Li + HCl → LiCl + H reaction at ⟨*E*_coll_⟩ = 0.132 eV (a) and ⟨*E*_coll_⟩ = 0.425 eV (c) that correspond to the nominal experimental collision energies of *E*_coll_ = 2.9 kcal mol^–1^ and *E*_coll_ = 9.2 kcal mol^–1^ (see ref (32)). (b, d) comparison between the experimental (solid symbols) and simulated LiCl LAB AD (blue line) using the QCT results at the indicated average collision energies. The simulated LAB AD was calculated as those in Figure 7.

**Figure 10:**
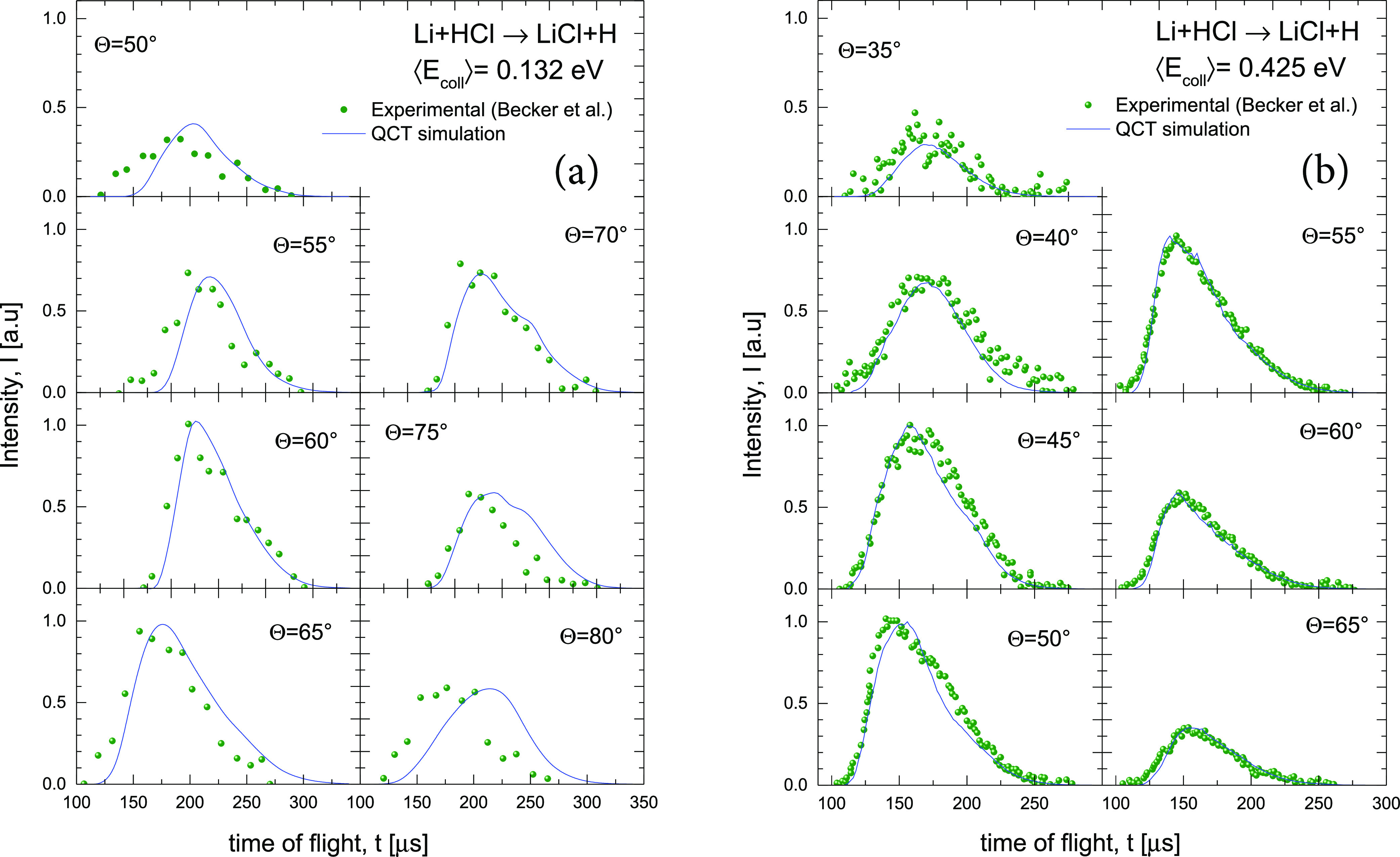
Comparison of experimental^32^ (solid symbols) and simulated (blue line) TOF distributions of the LiCl product at the indicated LAB angles and at ⟨*E*_coll_⟩ = 0.132 eV (a) and ⟨*E*_coll_⟩ = 0.425 eV (b), which correspond to the nominal experimental collision energies of 2.9 and 9.2 kcal mol^–1^, respectively.

The corresponding results for the Li + HCl reaction are shown in Figures 9 and 10. The polar maps, shown in panels (a) and (c) of Figure 9, are the TAV DCSs averaged over the collision energy and internal state distributions of the HCl beam. The contour plots can be compared with those shown in ref (32). At ⟨*E*_coll_⟩ = 0.132 eV, the scattering is almost entirely confined to the forward hemisphere, but spans a narrower cone than the experimentally derived counterpart. At ⟨*E*_coll_⟩ = 0.425 eV, scattering covers a wider range of angles than that shown in ref (32) but is restricted to a more reduced range of recoil velocities. The corresponding results, assuming that the angular and velocity distributions are not coupled, are shown in Figure S3 of the Supporting Information.

The theoretically simulated LAB ADs, shown in the right panels of Figure 9, are narrower than the experimental results, especially at low *E*_coll_. The agreement for ⟨*E*_coll_⟩ = 0.425 eV is better, but the simulation underestimates scattering at Θ_LAB_ lower than that of the CM. The TOF spectra are shown in Figure 10. The simulated TOFs have been scaled to the experimental ones for each spectrum to facilitate the comparison of the LAB velocity distributions at the different LAB angles. Therefore, they do not reflect the differences in magnitude found in the simulated LAB ADs. At the highest *E*_coll_, the scaling is ≈1 for most of the angles, especially for Θ_LAB_ ≥ 55°. At lower energy, the scaling differs from ≈1 only at Θ_LAB_ ≥ 70°. Although the overall agreement between the QCT simulations and the experimental LAB AD is significantly worse than for the Li + HF reaction, most of the features are well reproduced. The fact that the experimental LAB ADs are wider than the theoretically simulated ones can be traced back to the much wider recoil energy distributions in the experimental case (see panels (c) and (d) of Figure 6), which extend up to the thermodynamic limit. The authors of ref (32) admitted that it was not possible to improve the fit of the *I*_LAB_(Θ) without making the fit of the TOF data somewhat poorer.

**Figure 11:**
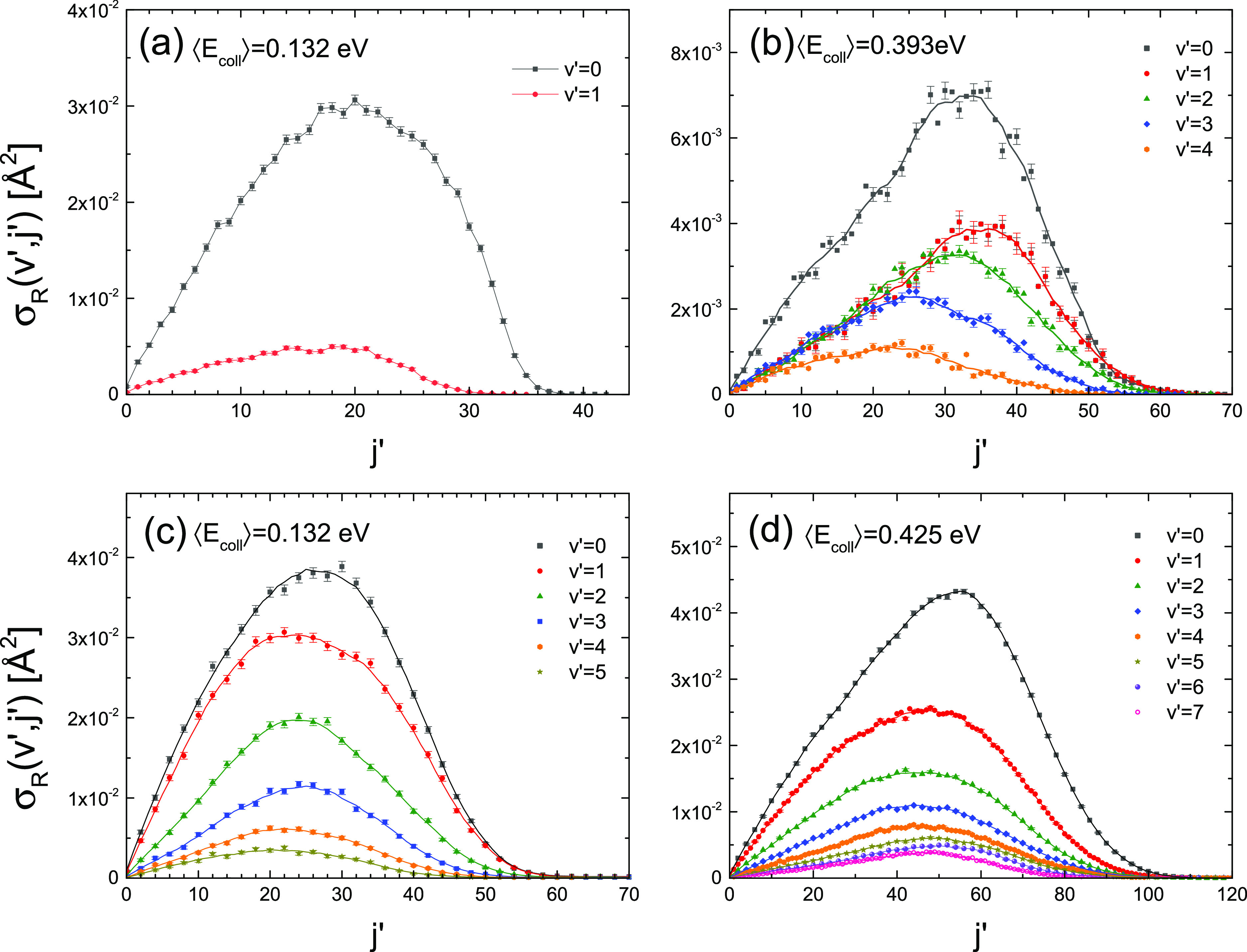
Rovibrational state distributions of LiF (a, b) and LiCl (c, d) reaction products calculated by QCT at the indicated average collision energies. The results have been averaged over the experimental collision and initial rotational energy distributions. The error bars represent twice the statistical uncertainties. The line through the points is a spline fit.

The QCT rovibrational distributions (ICS as a function of *j*′ for the various *v*′ manifolds) for the two reactions are shown in Figure 11. This figure simulates what would have been observed in the experiment with internal state resolution. The most salient result is that high rotational states are populated, their limiting value being caused by energy conservation. This is not surprising considering that the H + HL → HH + L kinematics lead to a complete  transfer. This issue will be discussed in the next section. The ICSs for Li + HF are shown in panels (a) and (b) for the two collision energies. At ⟨*E*_coll_⟩ = 0.132 eV, the LiF (*v*′ = 2, *j*′ = 0) state would be barely open. *v*′ = 1 is open, although the maximum *j*′ is overestimated in the QCT calculation. For ⟨*E*_coll_⟩ = 0.393 eV, LiF (*v*′ = 4, *j* > 35) would be barely open even if the spread of collision energy is taken into account. For the Li + HCl reaction (bottom panels), many more vibrational states can be energetically populated. The shapes of the rotational state distributions are similar to those for Li + HF at the highest collision energy.

## 5 Discussion

One of the main issues requiring an explanation is why the excitation functions for the two reactions are so different and yet their kinematics and their overall MEPs, shown in Figure 1, are similar. For Li + HF, the energy difference between the vibrationally adiabatic barrier and the ZPE of the HF is ≈0.065 eV. However, not only is no threshold found, but the cross section also increases with decreasing energy, as would be expected for a barrierless reaction. It is therefore clear that classical trajectories do not respect the ZPE of the TS. This implies that the reactant’s ZPE is used to overcome the barrier. No threshold is found in the QM case either. The deep vdW well prior to the barrier plays an important role. Under these circumstances, resonance-enhanced tunneling through the adiabatic barrier is probably the reason for the absence of a threshold in the quantum case. The sharp resonance structures found in the QM calculations below 0.1 eV support this interpretation.^33^ For Li + HCl, although it is lower, there is a threshold not only in the classical but also in the QM σ_R_.^60^ In this case, the energy difference between the vibrationally adiabatic barrier of the TS and the HCl ZPE is ≈0.061 eV, very similar to that of Li + HF. However, on the one hand, the barrier is somewhat broader (1400*i* cm^–1^ vs 1250*i* cm^–1^ for the LiHF TS), and on the other, the vdW well is very shallow. Therefore, resonance-enhanced tunneling is either absent or less favored, leading to a low threshold. While the entrance channel is mainly attractive in the Li + HF reaction, it is mainly repulsive in the Li + HCl system. In the classical case, the reaction coordinate is much less coupled to HCl vibration than in the case of Li + HF: not all of the vibrational energy of HCl is used to overcome the TS. It is sufficient to conserve the symmetric stretch to explain the observed threshold. This behavior is found in many reactions. For example, the H + H_2_ (*v* = 1) reaction has enough internal energy to overcome the barrier, but the symmetric stretch is not used, and there is a classical threshold.^78^

**Figure 12:**
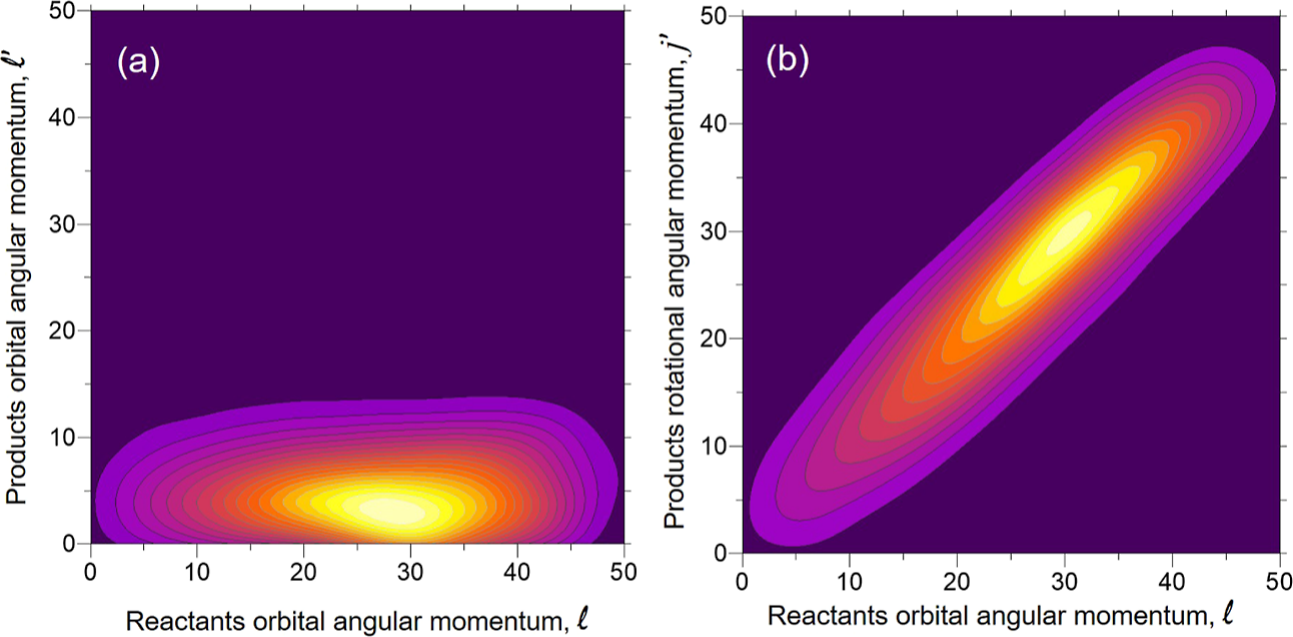
Contour map showing the correlation between the moduli of  (a) and  (b) for Li + HF (*v* = 0, *j* = 0) reaction at the fixed *E*_coll_ = 0.378 eV. Angular momenta in units of ℏ.

Another point that was discussed in the original article by Becker et al. is the correlation between the angular momenta of reactants and products. The results presented here are restricted to the *j* = 0 initial state of HF and HCl. The joint distributions of the moduli of  and  are shown in Figure 12a, and those of the respective moduli of  and ***j***′ are shown in Figure 12b for Li + HF at *E*_coll_ = 0.378 eV. There is only a weak correlation between ; the value of  is less than 10ℏ, and the most likely value is about 4ℏ. In contrast, there is a linear dependence of  with a central slope slightly less than one. The most probable values are  (*b* = 0.9 Å), which is the most probable value of  that will cause a reaction.

**Figure 13:**
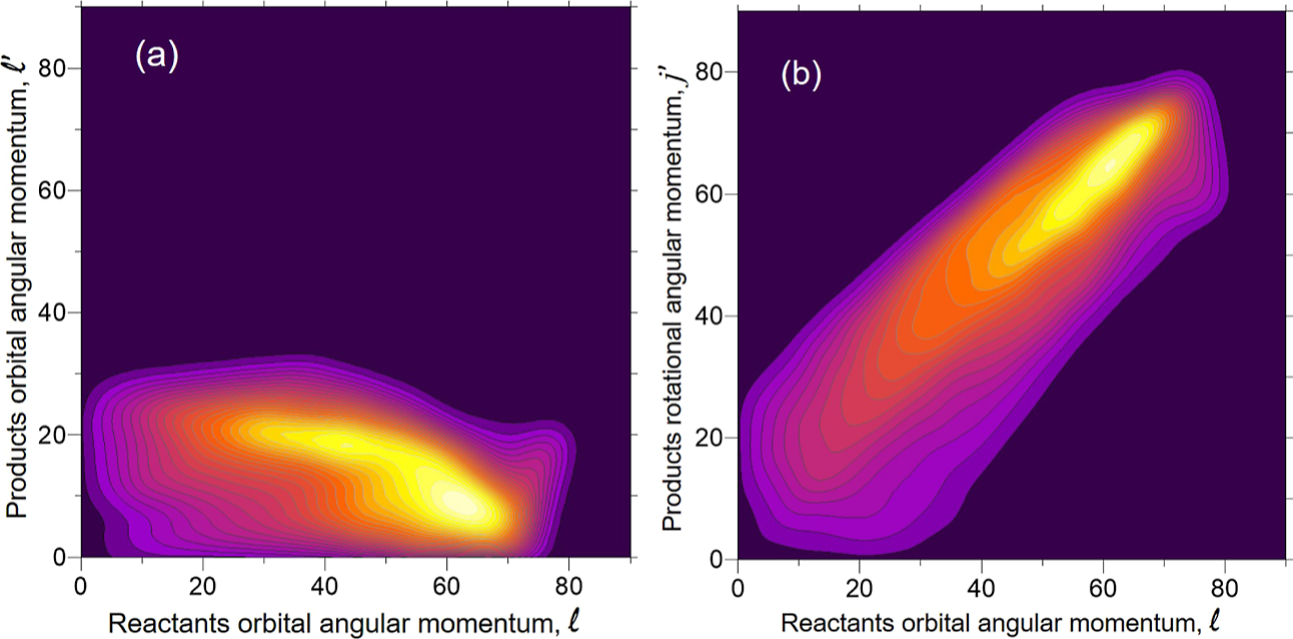
Same as Figure 12 but for the Li + HCl (*v* = 0, *j* = 0) reaction at fixed *E*_coll_ = 0.400 eV.

The analogous results for the Li + HCl (*v* = 0, *j* = 0) reaction are portrayed in Figure 13 for 0.400 eV collision energy. The correlations of the moduli of  show some interesting differences with respect to those in the Li + HF reaction. The joint distribution of  is broader, and larger values of  than for the Li + HF reaction can be attained. The most probable combination corresponds to . The kinematic constraint  is also satisfied with an average linear dependence. The distribution of  is broader than for Li + HF. At this collision energy, the highest value of  is 80ℏ (*b* = 2.4 Å), much lower than that assumed in the article by Becker et al. The most likely  is 60*ℏ* (*b* = 1.79 Å), which interestingly corresponds to a slightly larger *j*′ value.

**Figure 14:**
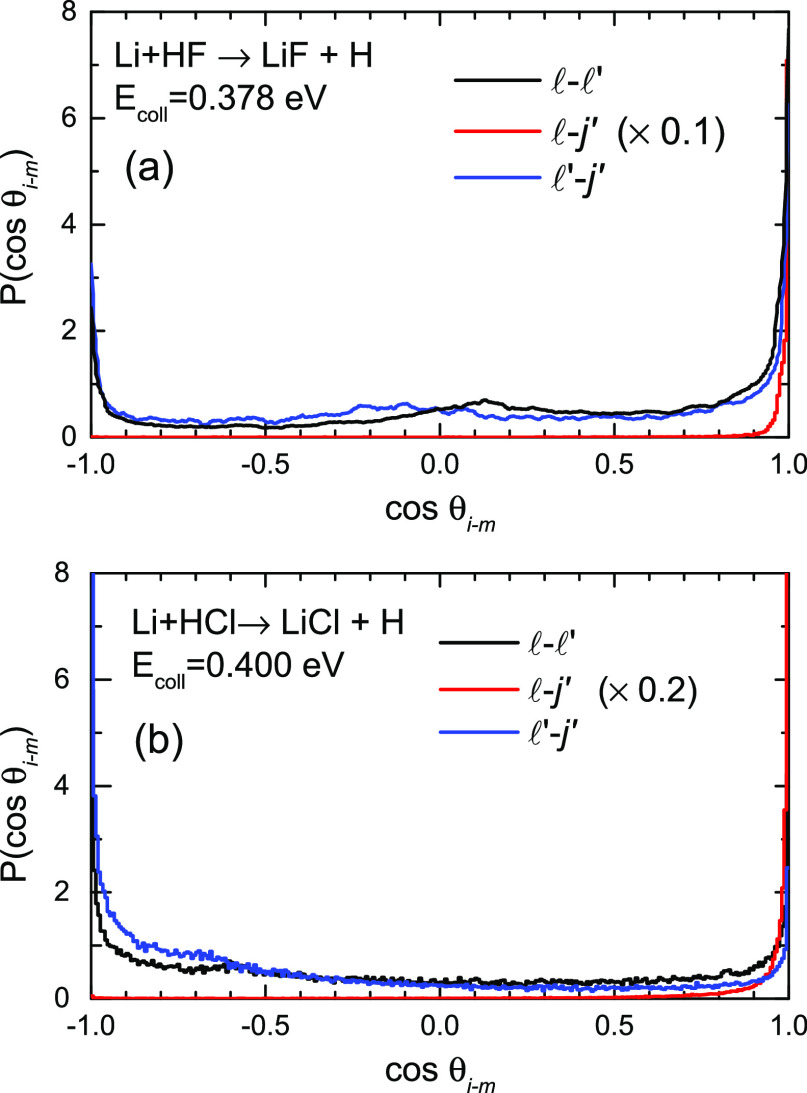
Classical vector correlations involving the directions of the initial orbital angular momentum, , the final rotational angular momentum, *j*′, and the final orbital angular momentum, , shown as the distributions of the cosine of the angles between these vectors: (a) Li + HF (*v* = 0, *j* = 0) at a fixed *E*_coll_ = 0.378 eV; (b) Li + HCl (*v* = 0, *j* = 0) at a fixed *E*_coll_ = 0.400 eV. As can be expected, ***j***′ is strongly parallel to .

The correlations between the directions of  are shown in Figure 14 for Li + HF panel (a) and for Li + HCl panel (b). For Li + HF, ***j***′ is strongly oriented along  (70% of the trajectories lie within a  interval). The product orbital angular momentum, , shows some preference to be parallel or antiparallel to . However, the calculations indicate an overall poor directional correlation between these two vectors. In the article by Becker et al., the authors speculate about the possible coplanarity of the reaction: if  were mainly aligned along  (parallel or antiparallel) the initial and final velocities would lie on the same plane. In addition, since ***j***′ is strongly oriented along , the outgoing LiF would rotate on the plane containing *v*_r_ and *v*_r_′ (cartwheel motion). The relatively poor correlation between  rules out coplanarity as the main mechanism, which would require  to be parallel or antiparallel. As expected, the correlation between  is similar to that of .

For the Li + HCl reaction at *E*_coll_ = 0.400 eV, the scenario is similar but with some interesting differences. Although ***j***′ mainly lies along , the distribution is slightly broader, within an angular cone of  (70% of the trajectories). In contrast,  are more correlated, with some preference for  to be antiparallel to . From the figure, it is clear that  has a preference to be antiparallel to ***j***′. These two facts explain that |***j***′| could be somewhat larger than , as discussed in relation to Figure 13b.

The formation of a long-lived complex in the Li + HF reaction at the lowest nominal collision energy of 0.130 eV was also discussed in ref (32). This assumption was based on the nearly symmetric backward–forward DCS at that energy. The present calculations do not predict such symmetry but a preference for forward scattering that, nevertheless, reproduces the LAB AD as long as the angular and velocity distributions are coupled. In any case, it is worthwhile to calculate the collision times at the two collision energies considered for each reaction.

**Figure 15:**
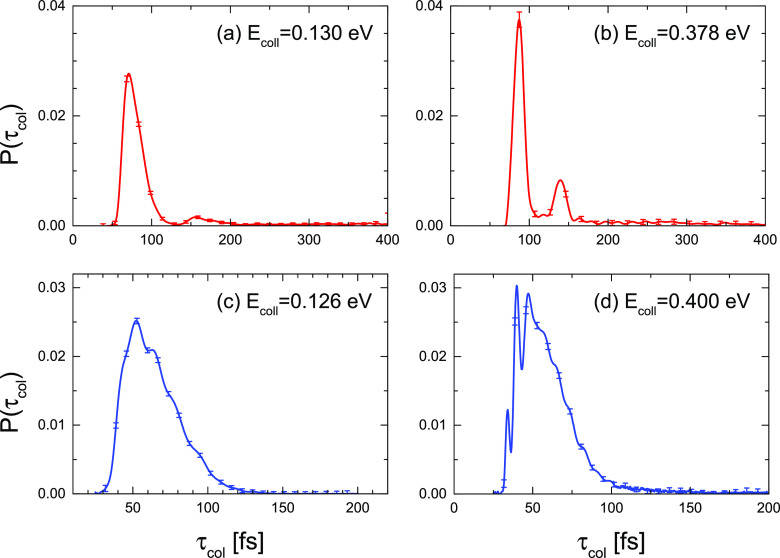
Distribution of collision times as defined in Section 3 for the reactions under study: (a, b) Li + HF (*v* = 0, *j* = 0) at 0.130 and 0.378 eV collision energies; (c, d) Li + HCl (*v* = 0, *j* = 0) at 0.126 and 0.400 eV collision energies.

Panels (a) and (b) of Figure 15 display the distribution of τ_coll_ at 0.130 and 0.378 eV nominal energies for the Li + HF (*v* = 0, *j* = 0) reaction. There are three main features that deserve some consideration. The first is that the distributions exhibit a sharp peak centered at ≈75 fs with most of the trajectories within τ_coll_ ≤ 100 fs. The second is that the distribution is bimodal, with a second peak of much less intensity and a larger τ_coll_. Finally, the presence of a long tail that extends beyond 200 fs with τ_coll_ that can reach 1 ps. More precisely, at 0.130 eV, ≈ 15% of the trajectories have τ_coll_ ≥ 220 fs. At 0.378 eV, the fraction is slightly lower ≈13.5%. Consequently, although the mechanism of a triatomic complex with a lifetime longer than the rotational period is far from predominant, there is a non-negligible fraction of trajectories with relatively long lifetimes. It is difficult to visualize the rotational motion of the complex, but inspection of movies of trajectories shows that the LiF remains nearly stationary while the H moves around.

Panels (c) and (d) of Figure 15 show the corresponding distributions of τ_coll_ at nominal energies of 0.126 and 0.400 eV for the Li + HCl (*v* = 0, *j* = 0) reaction. In contrast to the Li + HF reaction, only one peak is observed, which is similar in width to the faster one found in the Li + HF reaction, although slightly shifted to shorter collision times. No long tail is observed either. The distributions at the two collision energies display some structure, but it is difficult to assign specific characteristics to them. In spite of the presumed similarities between the two reactions, the one with HCl is a direct process with collision complexes of very short lifetimes.

**Figure 16:**
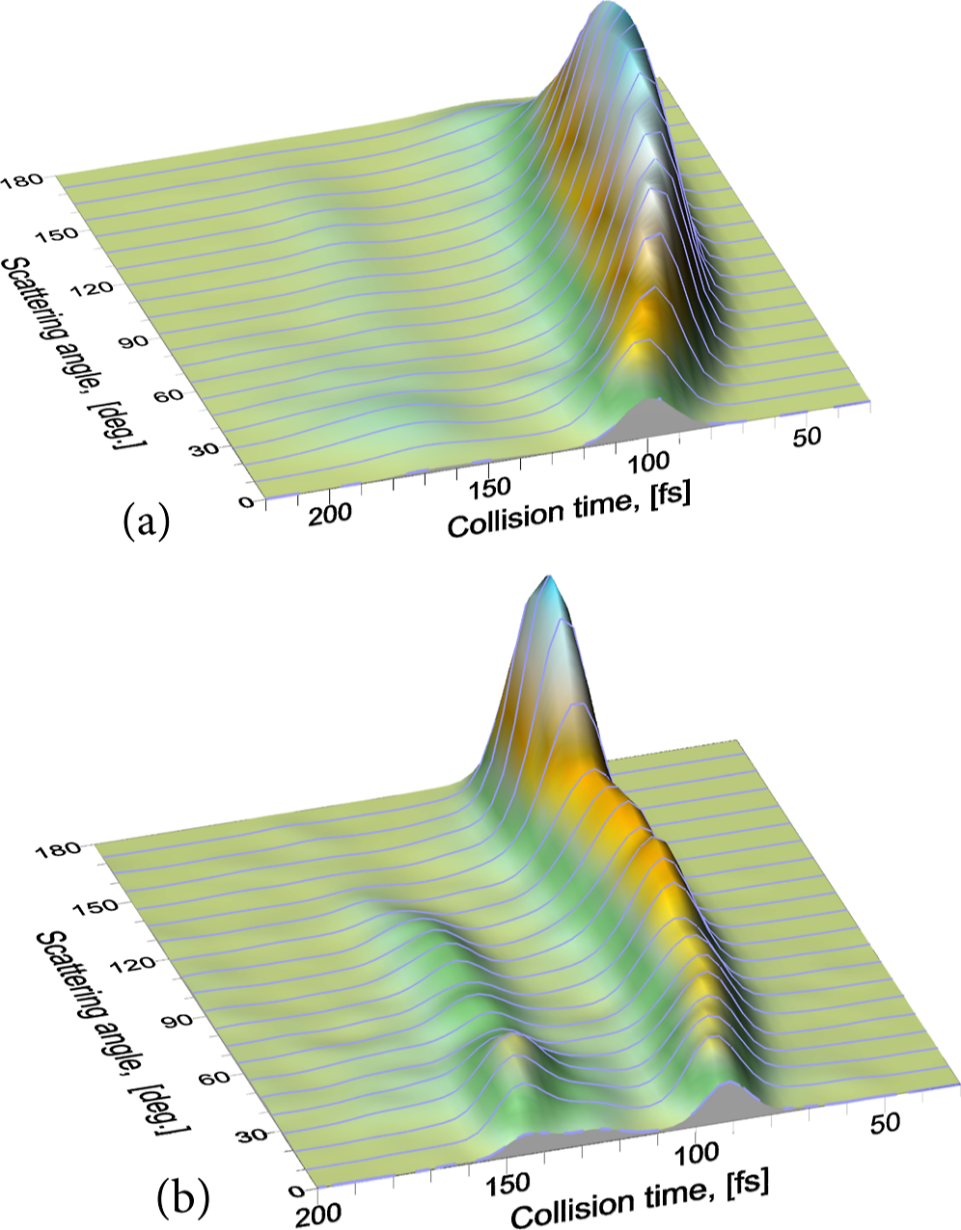
Joint distributions of collision times and scattering angles, *P*(τ_col_, θ) at 0.130 eV (a) and 0.378 eV (b) for the Li + HF reaction.

In the case of the Li + HF reaction, it remains to be seen whether the different peaks in the distribution of collision times correspond to specific scattering regions. In Figure 16, the joint *P*(τ_coll_, θ) is plotted for the two collision energies. At 0.130 eV, the first peak, which is dominant, comprises all the scattering angles. The observed ridge moves from backward scattering, with a slightly shorter τ_coll_, to forward scattering, where it peaks at 100 fs. The second maximum is barely visible and peaks at 170 fs, covering the whole angular range, although it seems to be more important at forward angles. The long tail discussed earlier is not visible in the figure. It seems to cover the whole range of scattering angles. At 0.378 eV, the contributions from the two peaks observed in the *P*(τ_col_) are clearly separated. The main contribution to the first peak can be clearly attributed to backscattering, although the ridge covers the whole angular range. Interestingly, the second peak, which appears at longer τ_coll_, has no contribution from backward scattering angles. Backward scattering (θ > 120°) is only contributed by collisions with short lifetimes, while sideways/forward scattering contains contributions from collisions with both short and long lifetimes and therefore show bimodal behavior.

## 6 Conclusions

The dynamics of the Li + HF and Li + HCl reactions have a long history since the pioneering and comprehensive work by Lee and co-workers 43 years ago. The original article aroused numerous theoretical and, to a lesser extent, experimental works. However, very few of the existing studies have paid attention to two important aspects. On the one hand, to the best of our knowledge, no simulation of the raw data (LAB ADs and TOF spectra) from the experiments of Becker et al. has been attempted until now. The comparison with the theoretical results has been limited to the experimentally derived DCSs and product recoil energy distributions obtained by forward convolution by trial and error fitting, assuming separability of the angular and recoil velocity distributions in the CM frame. On the other hand, the wealth of mechanistic information has been ignored without being critically examined.

Although accurate QM calculations for these reactions are becoming feasible and, in some instances, have been carried out for a limited range of collision energies and rotational states, the computational effort required to calculate the data necessary to simulate the raw experimental data is formidable. The choice of QCT calculations is judicious considering the large number of product states and the relatively low state resolution of the Becker et al. experiments. Therefore, in the present work, we have performed extensive QCT calculations on accurate PESs for each reaction in order to simulate, as closely as possible, the experimental data in the LAB system. This required the calculation of a large number of trajectories as a function of the collision energy and the initial rotational states for the Li + HF and Li + HCl reactions using the method that has been described in Section 3. Total cross sections, rovibrational distributions, DCSs, recoil energy distributions, opacity functions, etc., are among the quantities calculated for both reactions. In addition, aspects that were discussed in the article by Becker et al., such as the correlation between the angular momenta of reactants and products, or the possible formation of long-lived complexes, have been tackled in the present work.

To check the reliability of the QCT calculations, the QM CRP as a function of the total energy for total angular momentum *J* = 0 has been calculated and compared with that using the QCT approach. The agreement is very good, and the classical results can be considered a coarse-grained approximation to the QM results. This conclusion is supported by the good agreement between the present calculation of the total reaction cross section for different initial rotational states and those calculated on the same PESs (APW PES Li + HF and TZYGL PES for Li + HCl) using TI or TD-WP QM methods. Like their QM counterparts, the QCT calculations predict that σ_R_(*E*_coll_) for the Li + HF reaction has no threshold and decays rapidly with increasing *E*_coll_ to level off above 0.1 eV, in accordance with the experimental results at low energy. For Li + HCl, the QCT results predict an excitation function with a very low threshold (≤0.05 eV for *j* = 0), which grows rapidly with increasing *E*_coll_, reaching values one order of magnitude larger than for the Li + HF reaction at 0.5 eV. The agreement with the QM counterpart is also fairly good, although the QM calculations predict an even lower threshold.

QCT TAV DCSs have been calculated as a function of energy and for different initial rotational states. By averaging over the HX beam velocity distributions and the initial rotational state distributions, the corresponding polar maps have been constructed. The density-to-flux transformation from the CM to the LAB frame allows us to simulate the experimental LAB AD. Although the agreement with the LAB ADs and TOF spectra is very good for the Li + HF reaction, the theoretical and experimental DCS and recoil energy distributions in the CM differ significantly. The experimentally derived CM DCS and *P*(*E*_T_′) were obtained by trial-and-error fit of the LAB ADs and TOF spectra, assuming that the angular and recoil velocity distributions in the CM are separable. We have shown here that the calculated angle–recoil velocity polar maps in the CM differ from those assuming uncoupled distributions. This is the ultimate reason why there could be agreement in the LAB frame but significant disagreements in the assumed CM distributions.

For the Li + HCl reaction, the agreement is not so good, although the main aspects are well accounted for. The experimental LAB ADs are clearly broader than the simulated ones, although the TOF distributions, each of them scaled to its maximum, are in excellent agreement. It is tempting to attribute the discrepancies to the PES, but the present and earlier theoretical calculations on other PESs predict *P*(*E*_T_′) much narrower than those derived from the experiment. Analysis of the simulations shows that the origin of the wider LAB ADs lies in the difference in the *P*(*E*_T_′). The authors of ref (32) recognized that they could only fit the LAB ADs at the cost of a worse fit in the TOF distributions. In any case, further QM and QCT calculations on alternative PESs would be desirable.

The correlation between the angular momenta involved in the reaction has also been examined. The H + HL → HH + L kinematics constraint caused that  almost completely. However, against the coplanar mechanism proposed in the article by Becker et al., the direction of  is not necessarily aligned along the initial orbital angular momentum, which is the requirement for coplanarity. Finally, the distributions of collision times, the time that the three atoms spend together, show that both reactions are essentially direct, although about 15% of the LiFH complexes live longer than 200 fs. For this reaction, the calculated joint distribution of scattering angles and collision times indicates that there is a contribution to forward scattering from those trajectories that live for longer times.

It is difficult to categorize the two reactions, which also differ in many important dynamical aspects. Overall, they are direct reactions, with predominantly forward scattering, which share common features with reactions via harpooning, but the magnitude of impact parameters is much smaller. Li + HF also appears to share some aspects of barrierless reactions via collision complex formation, although the lifetime of the three-atom complex is much shorter. A more detailed study of the mechanism is required, and it will be the subject of a forthcoming article. On the experimental side, the use of new techniques with H detection instead of the LiX product, which is confined to a limited angular range in the LAB system, and the resolution in LiX internal states will provide an almost definitive understanding of these reactions.

## Supporting Information Available

(i) To illustrate the definition of collision time: Plot of the internuclear distances, *R*_HF_, *R*_LiF_, and *R*_LiH_, and the potential energy as a function of time for a reactive trajectory of the Li + HF reaction at *E*_coll_ = 0.378 eV; (ii) To show the differences between the actual angle-velocity polar maps (Figure 7) and those obtained with uncoupled distributions for the Li + HF reaction, at ⟨*E*_coll_⟩ = 0.132 eV (top) and ⟨*E*_coll_⟩ = 0.393 eV (bottom); (iii) The same but for the Li + HCl, Figure 9, at ⟨*E*_coll_⟩ = 0.132 eV (top) and ⟨*E*_coll_⟩ = 0.425 eV (bottom) (PDF)
