# Supplementary material for: Li + HF and Li + HCl Reactions Revisited I: QCT Calculations and Simulation of Experimental Results
Source: J Phys Chem A. 2023 Aug 14;127(33):6924–44. doi: 10.1021/acs.jpca.3c03763 (PMC10461305; doi:10.1021/acs.jpca.3c03763)
Supplement: Supplementary file 1 — jp3c03763_si_001.pdf [file jp3c03763_si_001.pdf]

Supplementary Information of: The Li+HF and Li+HCl Reactions Revisited I: QCT Calculations and Simulation of Experimental Results.

Marta Menéndez,[*,†] Ernesto Garcia,[‡] Manuel Lara,[¶] Pablo G. Jambrina,[§] and F. Javier Aoiz*[*,†]

†*Departamento de Química Física, Facultad de Ciencias Químicas. Universidad Complutense de Madrid , 28040 Madrid, Spain*

‡*Departamento de Química Física. Universidad del País Vasco (UPV/EHU), 01006 Vitoria, Spain*

¶*Departamento de Química Física Aplicada, Facultad de Ciencias. Universidad Autónoma de Madrid, 28039 Madrid, Spain*

§*Departamento de Química Física, Facultad de Ciencias Químicas. Universidad de Salamanca, 37008 Salamanca, Spain*

E-mail: menendez@quim.ucm.es; aoiz@quim.ucm.es

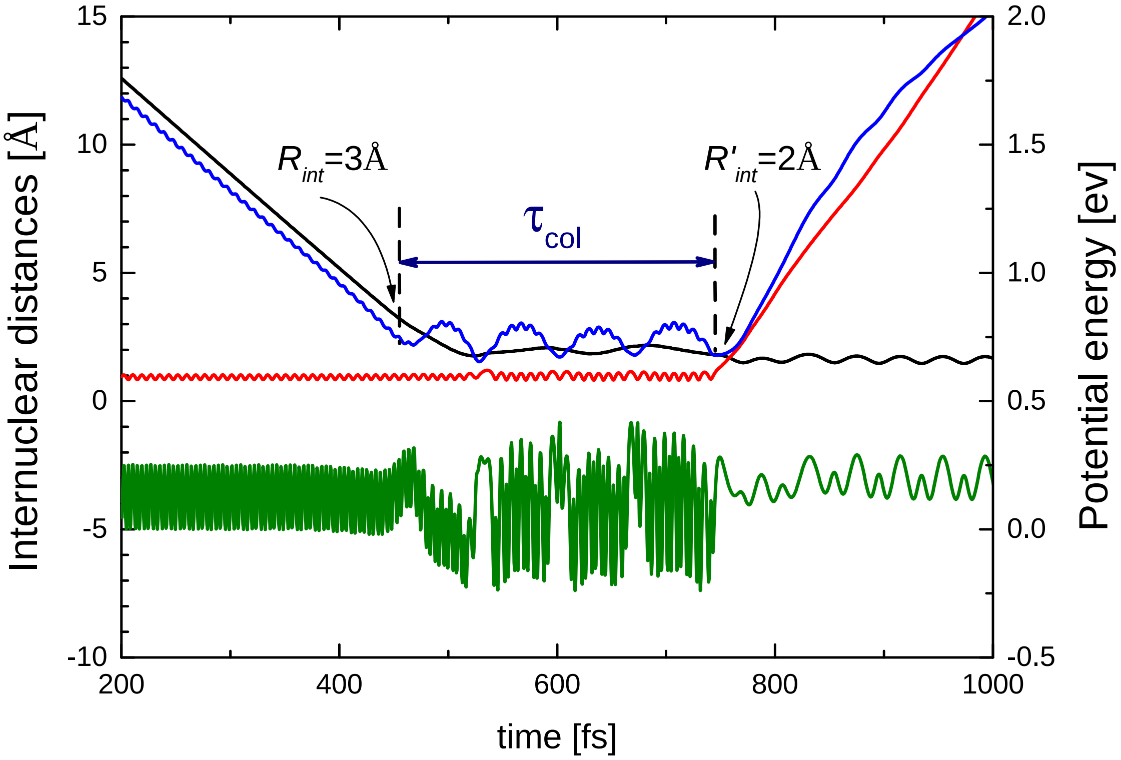

Figure S1. Plot of the internuclear distances, R_{HF} (red line), R_{LiF},(black line) and R_{LiH} (blue line), and the potential energy (green line) as a function of time for a reactive trajectory of the Li+HF reaction at E_{coll}=0.378 eV, to illustrate the definition of the collision time, τ_{coll}. R_{int} and R'_{int} are the initial CM $R_{\mathrm{Li-HF}}$ and the final $R_{\mathrm{H-LiF}}$ that define the strong interaction region. τ_{coll} is defined by the two vertical dashed lines. In this case its value is $\approx$ 300 fs.

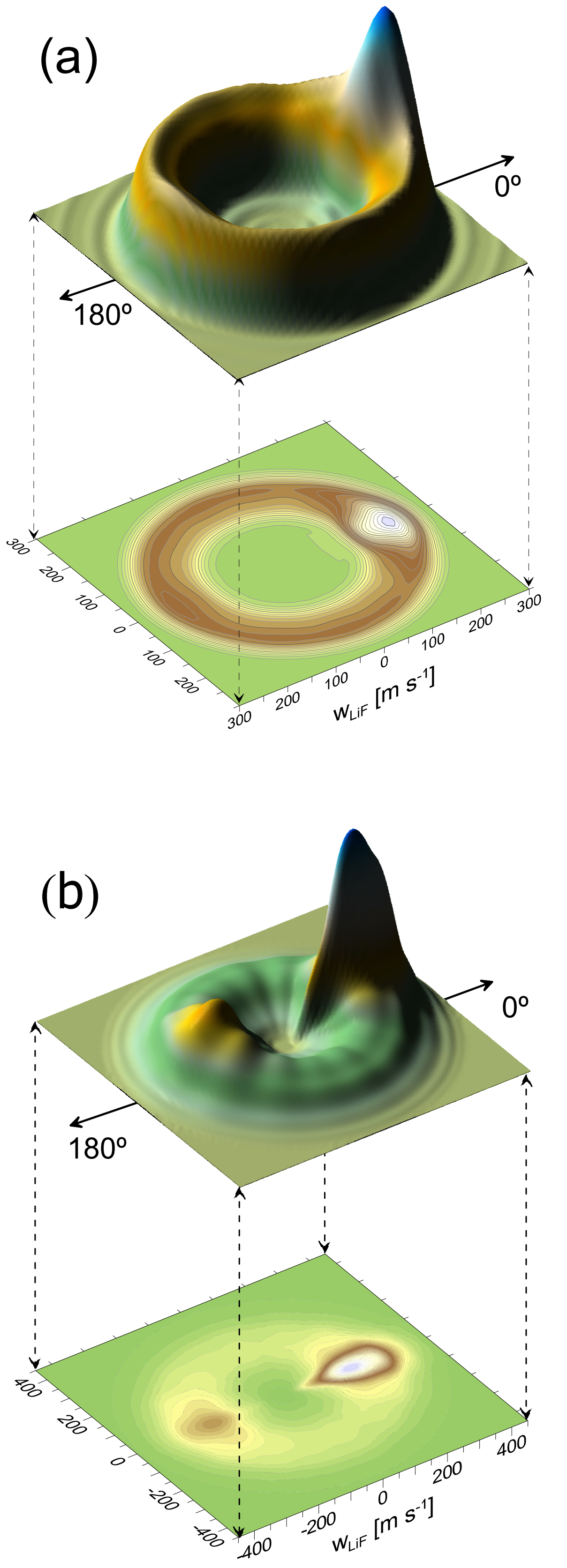

Figure S2. Three dimensional perspectives and contour polar plots of the uncoupled triple angle–velocity differential cross section the Li+HF reaction at 0.132 eV (a) and 0.393 eV (b) average collision energy.

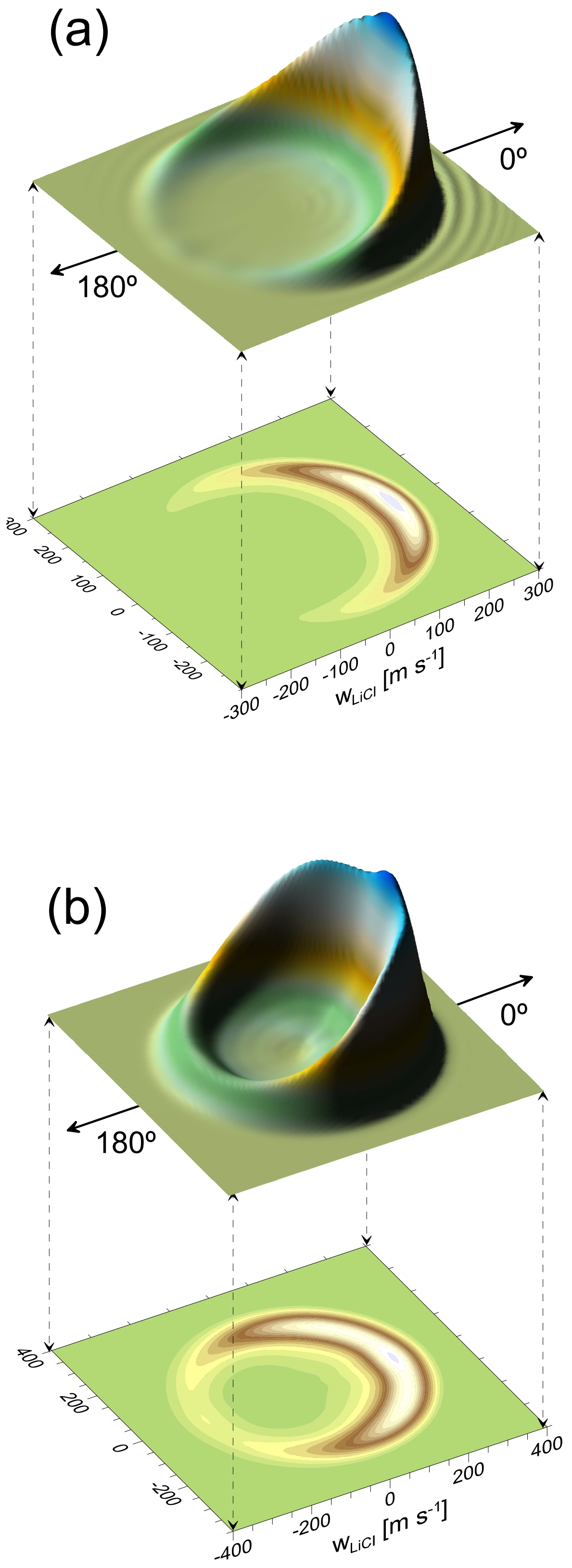

Figure S3. Three dimensional perspective and contour polar plots of the uncoupled triple angle–velocity differential cross section for the Li+ HCl reaction at (a) 0.132 eV and (b) 0.425 eV average collision energy.
